# Trends in views of democracy and society and support for political violence in the USA, 2022–2024: findings from a nationally representative survey

**DOI:** 10.1186/s40621-024-00550-0

**Published:** 2025-01-17

**Authors:** Garen J. Wintemute, Andrew Crawford, Elizabeth A. Tomsich, Veronica A. Pear

**Affiliations:** 1 UC Davis Violence Prevention Research Program, Sacramento, CA USA; 2 Department of Emergency Medicine, School of Medicine, UC Davis, Sacramento, CA USA; 3 California Firearm Violence Research Center, Sacramento, CA USA

**Keywords:** Political violence, Firearm violence, Violence and society, Racism, Domestic violent extremism, Civil war, QAnon

## Abstract

**Background:**

In 2022, a nationally representative longitudinal survey in the USA found concerningly high prevalences of support for and personal willingness to engage in political violence, but those prevalences decreased in 2023. This study examines changes in those prevalences from 2023 to 2024, an election year in the USA.

**Methods:**

Participants were members of Ipsos KnowledgePanel. Wave 3 of the survey was conducted May 23-June 14, 2024; invitations to participate were sent to all respondents to prior waves who remained in KnowledgePanel. Outcome measures concern justification for the use of violence to advance any of 17 specified political objectives, personal willingness to engage in political violence at 4 levels of severity and against 9 target populations, and expectation of firearm use in political violence. Outcomes are expressed as weighted proportions. Year-to-year change is based on the means of aggregated individual change scores, which have a potential range from 0 (no change) to ± 2.

**Results:**

The 2024 completion rates were 88.4% (8896 respondents/10,064 invitees) overall, 91.6% (8185 respondents/8932 invitees) for invitees in 2024 who had responded in 2023, and 62.8% (711 respondents/1132 invitees) for invitees in 2024 who had responded in 2022 but not in 2023. After weighting, 50.9% (95% confidence interval (CI) 49.5%, 52.3%) were female; weighted mean (SD) age was 48.5 (24.9) years. From 2023 to 2024, the prevalence of the view that violence was usually or always justified to advance at least 1 political objective did not change (2024: 26.2%, 95% CI 25.0%, 27.5%; 2023: 25.3%, 95% CI 24.1%, 26.5%). There were no changes from 2023 to 2024 in willingness to damage property, threaten a person, injure a person, or kill a person in an act of political violence, and no changes in expectations of firearm use in situations where respondents considered political violence justifiable. Changes on other measures were infrequent (17 of 58 comparisons in the main analysis) and small where they occurred (with 2 exceptions, change < 0.05).

**Conclusions:**

Contrary to expectation, support for and willingness to participate in political violence in this cohort showed little to no change from 2023 to 2024, an election year in the USA. These findings can help guide prevention efforts.

**Supplementary Information:**

The online version contains supplementary material available at 10.1186/s40621-024-00550-0.

## Background

Concern for the possibility of political violence in the USA has risen recently [1–8]. Experts have repeatedly stressed that such violence could threaten the health and safety of the population and the country’s viability as a functioning democracy.

In 2022, we conducted Wave 1 of a nationally representative longitudinal survey of support for and willingness to engage in political violence [9]. Nearly one-third of respondents (32.8%) considered violence usually or always justified to advance at least 1 of 17 specified political objectives; 13.7% strongly or very strongly agreed with a prediction of civil war in the next few years. These prevalences fell in 2023’s Wave 2, to 25.3% for justification of violence to advance specific political objectives and to 5.7% for an expectation of civil war [10]. While 2022 was an election year, 2023 was not; the declines were not surprising. Other findings from Waves 1 and 2 identified a broad array of respondent characteristics that were associated with support for and willingness to engage in political violence [11–15]. 

This study presents findings on support for political violence and many of those associated characteristics for 2024. It is motivated by our continued belief that understanding trends in support for and willingness to participate in political violence can strengthen efforts to prevent and prepare for that violence.

The value of trend data is in part a function of their recency. Wave 3 was in the field in May and June, less than 5 months before a strongly contested presidential election. A serial cross-sectional analysis of the Wave 3 data did not find an increase from 2023 to 2024 in a perceived need for civil war in the USA [16]. Nonetheless, our expectation for this analysis was that support for and willingness to engage in political violence would be higher in 2024 than in 2023.

The main analysis presents 2024 prevalences for all 8896 respondents and single-year changes from 2023 to 2024 based on linked observations for 8185 individuals who participated in both Wave 2 and Wave 3. Supplementary analyses address items that were presented only in 2023 and 2024, or only in 2022 and 2024. The survey was in the field at the time of Donald Trump’s 34 felony convictions in New York [17]; a sensitivity analysis compares responses received before and after that event.

## Methods

Methods for Wave 3 of the survey closely followed those for Waves 1 and 2 [9, 10]. Wave 3 was designed by the authors and administered online in English and Spanish from May 23 to June 14, 2024, by the survey research firm Ipsos [18]. The study was reviewed by the University of California Davis Institutional Review Board (protocol 187125: exempt from full review, category 2, survey research). The IRB waived a requirement for written or verbal consent. Before participants accessed the questionnaire, they were provided informed consent language that concluded, “[by] continuing, you are agreeing to participate in this study.” The study is reported following American Association for Public Opinion Research guidelines [19]. 

### Participants

Participants for Wave 1 were drawn from Ipsos KnowledgePanel, an online research panel that has been widely used in population-based research on violence and firearm ownership [20–25]. To establish a nationally representative panel, KnowledgePanel members are recruited on an ongoing basis through address-based probability sampling using data from the US Postal Service’s Delivery Sequence File [26, 27]. Recruitment into KnowledgePanel involves repeated contact attempts, if necessary, by mail and telephone. Recruited adults in households without internet access are provided a web-enabled device and free internet service, and a modest, primarily points-based incentive program seeks to encourage participation and promote participants’ retention in KnowledgePanel over time [26, 27]. 

A probability-proportional-to-size procedure was used to select a study-specific sample for Wave 1. All panel members who were aged 18 years and older were eligible for selection. Invitations were sent by e-mail; automatic reminders were delivered to non-respondents by e-mail and telephone beginning 3 days later [26, 27]. 

The Wave 1 survey was conducted May 13 to June 2, 2022. It included a main sample, which provided the study population for our initial report [9], and oversamples of firearm owners, transgender people, combat veterans, and California residents that were recruited to ensure adequate power for planned subset analyses. Compared with main sample nonrespondents, main sample respondents were older and more frequently white, non-Hispanic; were more often married; had higher education and income; and were less likely to be working [9]. 

The survey cohort’s participation history is presented in Figure S1 (Supplement, Additional file 1, Figure S1). Including the main sample and oversamples, Wave 1 comprised 12,947 respondents (completion rate of 56.7%). Of those respondents, 11,140 (86.0%) remained active members of KnowledgePanel on Wave 2’s launch date and were invited to participate in Wave 2. (The remaining 1807 Wave 1 respondents had left the cohort through normal attrition.)

Wave 2 had 9385 respondents (completion rate of 84.2%), of whom 8932 (95.2%) remained active members of KnowledgePanel on Wave 3’s launch date and were invited to participate in Wave 3. (Another 453 Wave 2 respondents had left the cohort through normal attrition.)

Invitations to participate in Wave 3 were also sent to 1132 Wave 1 respondents who had not participated in Wave 2 and remained active members of KnowledgePanel on Wave 3’s launch date. (Another 716 Wave 1 respondents who did not respond to Wave 2 had left the cohort through normal attrition.)

A final Wave 3 survey weight variable for longitudinal analyses was provided by Ipsos. It adjusted for the initial probability of selection into KnowledgePanel and for survey-specific nonresponse and over- or under-coverage using design weights with post-stratification raking ratio adjustments. As with prior samples, the weighted 2024 sample is designed to be statistically representative of the noninstitutionalized adult population of the USA as reflected in the 2021 March supplement of the Current Population Survey [26, 27]. 

### Measures

Sociodemographic data were collected by Ipsos from profiles created and maintained by KnowledgePanel members. Survey questions that supplied data for this analysis covered 3 broad domains: beliefs regarding democracy and the potential for violence and civil war in the USA, beliefs regarding American society and institutions, and support for and willingness to engage in political violence.

Our primary outcome measures again concerned political violence. Violence was represented by the phrase “force or violence,” defined in the questionnaire as “physical force strong enough that it could cause pain or injury to a person.” “Force or violence to advance an important political objective that you support” was used in questions about respondents’ support for and willingness to engage in political violence.

As in 2022 and 2023, respondents were asked about the extent to which they considered political violence to be justified “in general” and then about justification for its use to advance specified political objectives. Example objectives include “to return Donald Trump to the presidency this year,” “to preserve an American way of life based on Western European traditions,” and “to stop police violence.” Responses for 17 objectives were collected in all 3 years. In 2022, 9 of 17 objectives were presented to all respondents and 8 were paired, with respondents randomized for each pair to see 1 item; each respondent was presented with 13 of 17 objectives. In 2023 and 2024 all objectives were presented to all respondents; 2 additional objectives were included in 2023 and retained in 2024.

Respondents in 2024 who considered political violence to be at least sometimes justified to advance at least 1 objective were asked about their personal willingness to engage in political violence: by type of violence (to “damage property,” “threaten or intimidate a person,” “injure a person,” “kill a person”), by target population (examples: “an elected federal or state government official,” “a police officer,” “a person who does not share your religion”), and by social context (examples: “on your own,” “as part of a group”).

All respondents were asked about the likelihood of their future use of firearms in a situation where they consider political violence to be justified (examples: “I will be armed with a gun,” “I will shoot someone with a gun”).

The full text of all questions reported on here, including sources for questions from prior surveys by other investigators, is in the Supplement (Supplement, Additional File 1).

### Implementation

Ipsos translated the questionnaire into Spanish, and interpreting services staff at UC Davis Medical Center reviewed the translation. Twenty-three KnowledgePanel members participated in a pretest of the English language version that was administered May 10–14, 2024.

Respondents were randomized 1:1 to receive response options in order from either negative to positive valence (example: from ‘do not agree’ to ‘strongly agree’) or the reverse throughout the questionnaire. Where a question presented multiple statements for respondents to consider, the order in which those statements were presented was randomized unless ordering was necessary. Logic-driving questions (those to which responses might invoke a skip pattern) included non-response prompts.

We employed unipolar response arrays without a neutral midpoint (e.g., do not agree, somewhat agree, strongly agree, very strongly agree). The literature is not in agreement on whether such midpoints should be included [28, 29]. We were persuaded by the studies reviewed by Chyung et al. [28], which suggest that such midpoints allow respondents to choose “a minimally acceptable response as soon as it is found, instead of putting effort to find an optimal response,” a behavior known as satisficing. According to those authors, satisficing is particularly common when respondents are uncomfortable with the topics of the survey or under social desirability pressures, and both conditions apply here. Our analyses focus on responses above the “somewhat” or “sometimes” level to minimize the impact of potential satisficing on the results.

### Statistical analysis

IBM SPSS Statistics, version 29 (IBM Corp., Armonk, NY), was used for all analyses. Prevalence estimates were calculated as weighted percentages and 95% confidence intervals (CI) using Complex Samples Frequencies; mean differences and mean scores were calculated using Complex Samples Descriptives.

Each survey item was ordinal and was subject to non-response. We report weighted frequencies for each item for each possible response. In addition, we summarized each item’s non-missing responses for a given year by assigning integer values (1, 2, or 3) to ordinal levels to produce an item score and then averaging them.

The proportion of respondents reporting that violence was usually or always justified to advance at least 1 political objective was calculated in 2 ways. In the unrestricted version, the computation for each respondent was based on all objectives presented to that respondent in that year. In the restricted version, the computation for each respondent was based on the 13 objectives presented to that respondent in all 3 years.

To rigorously describe between-year changes in survey responses, we accounted for the longitudinal study design by computing within-individual change scores and then summarizing those. To compute differences in percentage choosing a particular response, we created indicator variables for each year for each item and each possible response and then computed the within-individual change score between the two survey years for each item and response level. To compute differences in mean response scores, we computed within-individual change scores for the item scores, restricted to the sample of respondents with non-missing responses to the item in both years. Year-to-year change is based on the means of aggregated individual change scores, which have a potential range from 0 (no change) to ± 2. We use the notation “change *x*, 95% CI *y*, *z*” [30] to report changes in mean scores.

The survey was in the field when Donald Trump was convicted on 34 felony charges in New York State Supreme Court at approximately 5 PM Eastern Daylight Time on May 30, 2024 [17]. We added a sensitivity analysis comparing responses on political violence items submitted before and after the convictions were announced.

## Results

The 2024 completion rates were 88.4% (8896 respondents/10,064 invitees) overall, 91.6% (8185 respondents/8932 invitees) for invitees in 2024 who had responded in 2023, and 62.8% (711 respondents/1132 invitees) for invitees in 2024 who had responded in 2022 but not in 2023. The median survey completion time for all Wave 3 respondents was 22 min (interquartile range, 15.7 min). Item non-response for items included in this analysis ranged from 0.4 to 3.1%; only 1 item had a non-response percentage above 3.0% (Supplement, Additional File 1).

After weighting, half of the respondents (50.9%, 95% CI 49.5%, 52.3%) were female; 62.7% (95% CI 61.2%, 64.2%) were white, non-Hispanic (Table 1). The weighted mean (SD) respondent age was 48.5 (24.9) years. Nonrespondents were younger than respondents (unweighted mean (SD) ages 53.8 (17.5) and 56.8 (16.5), respectively) and less frequently male and white, non-Hispanic (Supplement, Additional File 1, Table S1).

**Table 1:** Personal characteristics of respondents

Characteristic	2022 Respondents (*n*=12,947)		2023 Respondents* (*n*=9385)		2024 Respondents* (*n*=8896)	
	Unweighted *n*	Weighted % (95% CI)	Unweighted *n*	Weighted % (95% CI)	Unweighted *n*	Weighted % (95% CI)
Age						
18-24	488	10.5 (9.6, 11.4)	310	10.3 (9.2, 11.5)	176	6.6 (5.7, 7.7)
25-34	1309	16.4 (15.5, 17.4)	856	16.8 (15.6, 18.0)	753	17.4 (16.1, 18.7)
35-44	1884	18.5 (17.7, 19.4)	1252	18.5 (17.4, 19.6)	1094	16.9 (15.9, 18.1)
45-54	1847	14.5 (13.8, 15.2)	1255	14.3 (13.4, 15.2)	1150	14.9 (13.9, 15.9)
55-64	2794	17.5 (16.8, 18.2)	2043	17.6 (16.7, 18.5)	1827	18.4 (17.4, 19.4)
65-74	2952	14.4 (13.8, 15.1)	2342	14.5 (13.8, 15.3)	2249	15.1 (14.3, 15.8)
75+	1673	8.1 (7.6, 8.6)	1327	8.0 (7.4, 8.5)	1647	10.8 (10.1, 11.5)
Non-response	0	0.0 (0.0, 0.0)	0	0.0 (0.0, 0.0)	0	0.0 (0.0, 0.0)
Gender						
Female	5652	50.7 (49.6, 51.8)	3866	50.7 (49.4, 52.1)	3667	50.9 (49.5, 52.3)
Male	7028	47.2 (46.1, 48.3)	5340	47.0 (45.7, 48.4)	5055	47.5 (46.1, 48.9)
Transgender	74	0.5 (0.4, 0.7)	45	0.5 (0.3, 0.7)	46	0.5 (0.3, 0.8)
Non-binary	91	0.7 (0.5, 0.9)	59	0.8 (0.5, 1.0)	58	0.8 (0.6, 1.1)
Other	24	0.2 (0.1, 0.3)	21	0.3 (0.1, 0.5)	20	0.3 (0.2, 0.5)
Non-response	78	0.7 (0.5, 0.9)	54	0.7 (0.4, 0.9)	0	0.0 (0.0, 0.0)
Race/Ethnicity						
White, Non-Hispanic	9491	62.6 (61.5, 63.8)	7014	62.7 (61.2, 64.1)	6663	62.7 (61.2, 64.2)
Black, Non-Hispanic	1095	11.9 (11.1, 12.7)	748	12.0 (10.9, 13.0)	720	12.0 (11.0, 13.1)
Hispanic, any race	1504	16.9 (15.9, 17.8)	1016	16.9 (15.7, 18.1)	940	16.9 (15.7, 18.2)
American Indian or Alaska Native, Non-Hispanic	76	1.2 (0.8, 1.5)	47	1.1 (0.7, 1.5)	44	1.1 (0.7, 1.6)
Asian American or Pacific Islander, non-Hispanic	393	5.5 (4.8, 6.1)	277	5.5 (4.7, 6.2)	261	5.4 (4.7, 6.3)
Some other race, Non-Hispanic	25	0.1 (0.1, 0.2)	19	0.1 (0.1, 0.2)	18	0.1 (0.1, 0.2)
2+ Races, Non-Hispanic	363	1.8 (1.5, 2.0)	264	1.8 (1.4, 2.2)	250	1.8 (1.4, 2.2)
Non-response	0	0.0 (0.0, 0.0)	0	0.0 (0.0, 0.0)	0	0.0 (0.0, 0.0)
Marital status						
Now married	8074	56.1 (55.0, 57.3)	5961	56.2 (54.8, 57.6)	5655	57.0 (55.6, 58.5)
Widowed	770	4.1 (3.7, 4.5)	582	3.9 (3.5, 4.4)	630	4.7 (4.2, 5.2)
Divorced	1456	8.7 (8.2, 9.2)	1010	8.2 (7.6, 8.8)	979	8.5 (7.9, 9.2)
Separated	193	1.7 (1.4, 2.0)	122	1.4 (1.1, 1.8)	127	1.8 (1.4, 2.3)
Never married	2454	29.4 (28.2, 30.5)	1710	30.2 (28.8, 31.6)	1505	27.9 (26.5, 29.4)
Non-response	0	0.0 (0.0, 0.0)	0	0.0 (0.0, 0.0)	0	0.0 (0.0, 0.0)
Education						
No high school diploma or GED	624	9.4 (8.6, 10.2)	416	9.5 (8.4, 10.5)	330	7.4 (6.5, 8.4)
High school graduate (diploma, GED)	2813	28.2 (27.2, 29.3)	2002	28.2 (26.9, 29.6)	1784	26.9 (25.5, 28.2)
Some college or Associate’s degree	3896	27.2 (26.2, 28.1)	2773	27.1 (25.9, 28.3)	2691	28.7 (27.5, 30.0)
Bachelor’s degree	3133	19.8 (19.0, 20.6)	2337	20.1 (19.1, 21.1)	2257	21.0 (19.9, 22.1)
Master’s degree or higher	2481	15.4 (14.7, 16.1)	1857	15.1 (14.2, 15.9)	1834	16.0 (15.2, 17.0)
Non-response	0	0.0 (0.0, 0.0)	0	0.0 (0.0, 0.0)	0	0.0 (0.0, 0.0)
Household Income						
Less than $10,000	371	3.9 (3.4, 4.4)	233	3.9 (3.2, 4.5)	265	4.9 (4.2, 5.7)
$10,000 to $24,999	1078	9.0 (8.3, 9.6)	727	8.9 (8.1, 9.8)	609	7.9 (7.1, 8.7)
$25,000 to $49,999	2232	17.0 (16.2, 17.9)	1617	17.0 (15.9, 18.0)	1446	17.1 (16.0, 18.3)
$50,000 to $74,999	2236	16.3 (15.5, 17.2)	1631	16.3 (15.3, 17.4)	1424	15.3 (14.3, 16.3)
$75,000 to $99,999	1999	13.2 (12.5, 13.9)	1499	13.2 (12.3, 14.1)	1367	13.3 (12.4, 14.3)
$100,000 to $149,999	2410	17.9 (17.0, 18.7)	1734	17.9 (16.8, 18.9)	1799	18.5 (17.5, 19.6)
$150,000 or more	2621	22.7 (21.7, 23.6)	1944	22.8 (21.6, 23.9)	1986	23.0 (21.9, 24.2)
Non-response	0	0.0 (0.0, 0.0)	0	0.0 (0.0, 0.0)	0	0.0 (0.0, 0.0)
Employment						
Working - as a paid employee	6213	53.8 (52.7, 54.9)	4291	52.9 (51.6, 54.3)	4134	53.2 (51.8, 54.7)
Working - self-employed	1048	8.0 (7.4, 8.6)	709	7.2 (6.5, 8.0)	669	7.2 (6.6, 8.0)
Not working - on temporary layoff from a job	53	0.6 (0.4, 0.8)	35	0.5 (0.3, 0.7)	37	0.6 (0.4, 0.9)
Not working - looking for work	411	5.2 (4.6, 5.8)	272	5.2 (4.4, 5.9)	251	4.9 (4.2, 5.7)
Not working - retired	4231	21.0 (20.3, 21.8)	3367	21.3 (20.4, 22.2)	3154	21.4 (20.5, 22.4)
Not working - disabled	417	4.2 (3.7, 4.7)	286	4.5 (3.9, 5.2)	258	4.4 (3.8, 5.1)
Not working - other	574	7.2 (6.6, 7.9)	425	8.3 (7.4, 9.2)	393	8.2 (7.3, 9.1)
Non-response	0	0.0 (0.0, 0.0)	0	0.0 (0.0, 0.0)	0	0.0 (0.0, 0.0)
Census division						
New England	509	4.7 (4.2, 5.2)	374	4.7 (4.1, 5.3)	362	4.6 (4.1, 5.3)
Mid-Atlantic	1407	12.5 (11.8, 13.3)	1001	12.6 (11.6, 13.5)	960	12.4 (11.5, 13.4)
East-North Central	1878	14.3 (13.5, 15.0)	1370	14.3 (13.3, 15.2)	1306	14.3 (13.4, 15.3)
West-North Central	952	6.4 (5.9, 6.9)	676	6.4 (5.8, 7.0)	647	6.4 (5.8, 7.1)
South Atlantic	2538	20.5 (19.6, 21.4)	1881	20.5 (19.4, 21.6)	1754	20.6 (19.4, 21.8)
East-South Central	737	5.8 (5.3, 6.3)	538	5.8 (5.1, 6.5)	514	5.9 (5.3, 6.6)
West-South Central	1371	11.9 (11.1, 12.7)	965	11.9 (10.9, 12.8)	902	11.7 (10.8, 12.7)
Mountain	1125	7.7 (7.1, 8.2)	825	7.6 (6.9, 8.3)	796	7.7 (7.0, 8.5)
Pacific	2430	16.3 (15.5, 17.1)	1755	16.3 (15.3, 17.3)	1655	16.3 (15.3, 17.4)
Non-response	0	0.0 (0.0, 0.0)	0	0.0 (0.0, 0.0)	0	0.0 (0.0, 0.0)

Findings for 2022 and 2023 respondents were published previously [10] and are reproduced for convenience

* Most values are as of 2022; census division values were updated for 2024, but other demographics were not

### Democracy and the potential for violence

There were small but consistent increases in pro-democracy views from 2023 to 2024 (Table 2): increases in the view that it is very or extremely important for the United States to remain a democracy (change 0.040, 95% CI 0.024, 0.055) and that democracy is the best form of government (change 0.033, 95% CI 0.014, 0.053), and decreased support for the positions that democracy only serves the interests of the wealthy and powerful (change − 0.107, 95% CI -0.131, -0.083) and that having a strong leader is more important than having a democracy (change − 0.024, 95% CI -0.048, -0.001).

**Table 2:** Beliefs concerning democracy in the United States

Statement	2022 Respondents (*n*= 12,947)		2023 Respondents (*n*=9385)		2024 Respondents (*n*=8896)		Mean Difference, * 2022-2023		Mean Difference, * 2023-2024	
	Unweighted *n*	Weighted % (95% CI) Mean score (95% CI)	Unweighted *n*	Weighted % (95% CI) Mean score (95% CI)	Unweighted *n*	Weighted % (95% CI) Mean score (95% CI)	Unweighted *n*	Weighted % (95% CI) Mean score (95% CI)	Unweighted *n*	Weighted % (95% CI) Mean score (95% CI)
When thinking about democracy in the United States these days, do you believe…										
There is a serious threat to our democracy. (1)	9409	67.4 (66.3, 68.5)	6452	62.3 (60.9, 63.7)	6256	64.8 (63.4, 66.2)	9385	− 5.2 (− 6.6, − 3.8)	8185	1.3 (− 0.2, 2.7)
There may be a threat to our democracy, but it is not serious. (2)	2640	23.5 (22.5, 24.5)	2253	28.0 (26.8, 29.3)	1953	25.9 (24.6, 27.2)	9385	4.7 (3.3, 6.2)	8185	− 1.5 (− 3.0, 0.1)
There is no threat to our democracy. (3)	780	7.7 (7.0, 8.4)	529	7.0 (6.2, 7.8)	573	7.3 (6.5, 8.1)	9385	− 0.7 (− 1.8, 0.3)	8185	0.6 (− 0.3, 1.6)
Non-response	118	1.4 (1.1, 1.7)	151	2.6 (2.1, 3.1)	114	2.0 (1.6, 2.5)	9385	1.2 (0.7, 1.8)	8185	− 0.4 (− 0.9, 0.0)
* Item score†*	*12,829*	*1.39 (1.38*,* 1.41)*	*9234*	*1.43 (1.41*,* 1.45)*	*8782*	*1.41 (1.39*,* 1.43)*	*9194*	*0.041 (0.022*,* 0.061)*	*8020*	*− 0.007 (− 0.026*,* 0.012)*
How important do you think it is for the United States to remain a democracy?										
Not important (1)	191	2.1 (1.8, 2.5)	261	4.0 (3.4, 4.6)	193	3.5 (2.9, 4.2)	9385	1.8 (1.2, 2.5)	8185	− 0.7 (− 1.5, 0.0)
Somewhat important (2)	659	7.7 (7.0, 8.4)	570	9.7 (8.8, 10.7)	411	7.6 (6.8, 8.5)	9385	2.2 (1.2, 3.3)	8185	− 2.1 (− 3.3, -1.0)
Very or extremely important (3)	12,003	89.0 (88.2, 89.8)	8448	84.6 (83.4, 85.7)	8208	87.5 (86.3, 88.5)	9385	− 4.6 (− 5.6, − 3.5)	8185	2.9 (1.7, 4.0)
Non-response	94	1.2 (0.9, 1.4)	106	1.7 (1.3, 2.2)	84	1.4 (1.1, 1.9)	9385	0.5 (0.0, 0.9)	8185	0.0 (− 0.4, 0.3)
* Item score†*	*12,853*	*2.88 (2.87*,* 2.89)*	*9279*	*2.82 (2.80*,* 2.84)*	*8812*	*2.85 (2.84*,* 2.87)*	*9241*	*− 0.064 (− 0.078*,* − 0.051)*	*8071*	***0.040 (0.024***,*** 0.055)***
Democracy is the best form of government.										
Do not agree (1)	595	5.8 (5.2, 6.4)	531	7.5 (6.7, 8.4)	493	7.2 (6.4, 8.0)	9385	2.0 (1.1, 2.9)	8185	− 0.8 (− 1.8, 0.2)
Somewhat agree (2)	2396	23.1 (22.1, 24.1)	1765	24.1 (22.8, 25.3)	1507	22.0 (20.7, 23.3)	9385	1.1 (− 0.4, 2.5)	8185	− 1.7 (− 3.3, − 0.1)
Strongly or very strongly agree (3)	9823	69.5 (68.5, 70.6)	6948	65.9 (64.5, 67.3)	6775	68.7 (67.3, 70.1)	9385	− 3.9 (− 5.3, − 2.5)	8185	2.5 (1.1, 4.0)
Non-response	133	1.6 (1.3, 1.9)	141	2.5 (2.0, 3.0)	121	2.2 (1.7, 2.7)	9385	0.8 (0.3, 1.3)	8185	− 0.1 (− 0.5, 0.4)
Item score†	*12,814*	*2.65 (2.63*,* 2.66)*	*9244*	*2.60 (2.58*,* 2.62)*	*8775*	*2.63 (2.61*,* 2.65)*	*9191*	*− 0.057 (− 0.075*,* − 0.039)*	*8029*	***0.033 (0.014***,*** 0.053)***
These days, American democracy only serves the interest of the wealthy and powerful.										
Do not agree (1)	3976	26.3 (25.4, 27.2)	2789	25.8 (24.6, 26.9)	3301	31.8 (30.6, 33.1)	9385	− 1.0 (− 2.4, 0.3)	8185	6.4 (5.0, 7.9)
Somewhat agree (2)	4499	36.1 (35.0, 37.2)	3678	39.6 (38.2, 40.9)	3239	37.3 (35.9, 38.6)	9385	3.3 (1.6, 5.0)	8185	− 2.3 (− 4.1, − 0.5)
Strongly or very strongly agree (3)	4354	36.2 (35.1, 37.3)	2781	32.2 (30.9, 33.5)	2259	29.2 (27.9, 30.6)	9385	− 3.2 (− 4.7, -1.7)	8185	− 3.6 (− 5.1, − 2.0)
Non-response	118	1.4 (1.1, 1.7)	137	2.4 (1.9, 3.0)	97	1.7 (1.3, 2.2)	9385	0.9 (0.5, 1.4)	8185	− 0.6 (− 1.0, − 0.1)
* Item score†*	*12,829*	*2.10 (2.08*,* 2.12)*	*9248*	*2.07 (2.04*,* 2.09)*	*8799*	*1.97 (1.95*,* 2.00)*	*9199*	*− 0.020 (− 0.044*,* 0.003)*	*8036*	***− 0.107 (− 0.131***,*** − 0.083)***
Having a strong leader for America is more important than having a democracy.										
Do not agree (1)	7921	56.2 (55.1, 57.3)	6219	59.6 (58.2, 61.0)	6076	63.0 (61.6, 64.4)	9385	3.0 (1.6, 4.4)	8185	2.7 (1.2, 4.2)
Somewhat agree (2)	2628	23.0 (22.1, 24.0)	1685	21.7 (20.5, 22.9)	1403	18.8 (17.6, 20.0)	9385	− 1.5 (− 3.1, 0.0)	8185	− 2.8 (− 4.4, -1.2)
Strongly or very strongly agree (3)	2254	19.1 (18.2, 20.0)	1333	16.1 (15.0, 17.1)	1280	15.9 (14.9, 17.1)	9385	− 2.3 (− 3.6, -1.1)	8185	0.3 (− 1.0, 1.6)
Non-response	144	1.6 (1.3, 2.0)	148	2.6 (2.1, 3.2)	137	2.3 (1.8, 2.8)	9385	0.8 (0.3, 1.3)	8185	− 0.2 (− 0.7, 0.3)
* Item score†*	*12,803*	*1.62 (1.60*,* 1.64)*	*9237*	*1.55 (1.53*,* 1.57)*	*8759*	*1.52 (1.50*,* 1.54)*	*9182*	*− 0.057 (− 0.079*,* − 0.035)*	*8001*	***− 0.024 (− 0.048***,*** − 0.001)***
The 2020 election was stolen from Donald Trump, and Joe Biden is an illegitimate president.										
Do not agree (1)	8442	66.9 (65.8, 67.9)	6135	66.7 (65.4, 68.0)	5843	66.7 (65.3, 68.0)	9385	− 1.0 (− 1.9, 0.0)	8185	− 0.6 (− 1.6, 0.4)
Somewhat agree (2)	1830	13.5 (12.8, 14.3)	1364	14.1 (13.1, 15.1)	1338	14.3 (13.4, 15.4)	9385	1.0 (0.0, 2.1)	8185	0.6 (− 0.5, 1.7)
Strongly or very strongly agree (3)	2502	17.9 (17.0, 18.7)	1729	16.7 (15.7, 17.7)	1580	17.0 (16.0, 18.1)	9385	− 0.9 (− 1.8, 0.0)	8185	0.2 (− 0.8, 1.1)
Non-response	173	1.7 (1.4, 2.0)	157	2.5 (2.0, 3.0)	135	2.0 (1.6, 2.4)	9385	0.9 (0.4, 1.4)	8185	− 0.2 (− 0.7, 0.3)
* Item score†*	*12,774*	*1.50 (1.48*,* 1.52)*	*9228*	*1.49 (1.47*,* 1.51)*	*8761*	*1.49 (1.47*,* 1.52)*	*9164*	*0.001 (− 0.014*,* 0.015)*	*7995*	*0.006 (− 0.009*,* 0.022)*

Change scores have a potential range from 0 (no change) to ±2. Change scores for 2023-2034 that differ significantly from 0 are in bold font. Findings for 2022 and 2023 respondents and mean differences for 2022-2023 were published previously [10] and are reproduced for convenience

* Among respondents to both surveys

† Mean scores were calculated using values indicated in the response lines for individual items. Non-responses were excluded from mean score calculations and differences in mean scores were computed in the subsample of respondents with non-missing responses in both years by computing within-individual change scores and averaging them, to account for the longitudinal study design. For computing differences in individual response levels, indicator variables were computed for each item for each response level and within-individual differences in these were computed and averaged in the subsample of respondents who responded to the survey in both years. This explains the variation in the unweighted n for the mean differences

There was no change from 2023 to 2024 in support for 3 statements about conditions in the USA justifying force or violence (Table 3). There was a small increase in expectation of civil war in the USA in the next few years (change 0.026, 95% CI 0.007, 0.045).

**Table 3:** Beliefs concerning the potential need for violence in the United States

Statement	2022 Respondents (*n*= 12,947)		2023 Respondents (*n*=9385)		2024 Respondents (*n*=8896)		Mean Difference, * 2022-2023		Mean Difference, * 2023-2024	
	Unweighted *n*	Weighted % (95% CI) Mean score (95% CI)	Unweighted *n*	Weighted % (95% CI) Mean score (95% CI)	Unweighted *n*	Weighted % (95% CI) Mean score (95% CI)	Unweighted *n*	Weighted % (95% CI) Mean score (95% CI)	Unweighted *n*	Weighted % (95% CI) Mean score (95% CI)
If elected leaders will not protect American democracy, the people must do it themselves, even if it requires taking violent actions.										
Do not agree (1)	6461	50.2 (49.1, 51.3)	5920	62.1 (60.7, 63.4)	5546	62.4 (61.0, 63.8)	9385	11.6 (10.1, 13.0)	8185	0.1 (− 1.4, 1.6)
Somewhat agree (2)	3838	29.6 (28.6, 30.6)	2397	25.9 (24.7, 27.2)	2310	24.7 (23.5, 26.0)	9385	− 3.3 (− 4.9, -1.7)	8185	− 1.4 (− 3.0, 0.1)
Strongly or very strongly agree (3)	2502	18.5 (17.6, 19.4)	919	9.7 (8.8, 10.5)	901	10.3 (9.5, 11.3)	9385	− 8.8 (− 10.0, − 7.7)	8185	0.8 (− 0.2, 1.8)
Non-response	146	1.6 (1.3, 2.0)	149	2.4 (1.9, 2.8)	139	2.6 (2.1, 3.2)	9385	0.6 (0.1, 1.1)	8185	0.5 (0.0, 1.0)
* Item score†*	*12,801*	*1.68 (1.66*,* 1.69)*	*9236*	*1.46 (1.44*,* 1.48)*	*8757*	*1.47 (1.45*,* 1.49)*	*9170*	*− 0.211 (− 0.233*,* − 0.190)*	*8003*	*0.006 (− 0.015*,* 0.026)*
Our American way of life is disappearing so fast that we may have to use force to save it.										
Do not agree (1)	7360	56.0 (54.9, 57.1)	5733	59.6 (58.2, 61.0)	5583	61.9 (60.5, 63.3)	9385	3.3 (1.9, 4.7)	8185	− 0.1 (− 1.4, 1.3)
Somewhat agree (2)	3406	26.7 (25.7, 27.7)	2419	26.0 (24.8, 27.3)	2184	23.7 (22.5, 24.9)	9385	− 0.3 (− 1.8, 1.3)	8185	− 0.9 (− 2.4, 0.5)
Strongly or very strongly agree (3)	2032	15.8 (15.0, 16.6)	1101	12.1 (11.1, 13.0)	1005	12.1 (11.1, 13.1)	9385	− 3.6 (− 4.7, − 2.4)	8185	0.5 (− 0.5, 1.6)
Non-response	149	1.5 (1.2, 1.8)	132	2.3 (1.8, 2.8)	124	2.3 (1.8, 2.8)	9385	0.6 (0.1, 1.1)	8185	0.4 (0.0, 0.9)
* Item score†*	*12,798*	*1.59 (1.58*,* 1.61)*	*9253*	*1.51 (1.49*,* 1.53)*	*8772*	*1.49 (1.47*,* 1.51)*	*9182*	*− 0.071 (− 0.092*,* − 0.050)*	*8018*	*0.005 (− 0.014*,* 0.025)*
Because things have gotten so far off track, true American patriots may have to resort to violence in order to save our country.										
Do not agree (1)	9486	72.6 (71.6, 73.6)	6905	71.6 (70.3, 72.9)	6578	72.1 (70.7, 73.4)	9385	− 1.4 (− 2.7, − 0.1)	8185	1.5 (0.1, 3.0)
Somewhat agree (2)	2287	17.8 (16.9, 18.6)	1675	18.5 (17.4, 19.6)	1539	17.4 (16.3, 18.5)	9385	1.3 (0.0, 2.6)	8185	− 1.8 (− 3.4, − 0.3)
Strongly or very strongly agree (3)	992	7.7 (7.1, 8.3)	670	7.6 (6.8, 8.4)	642	8.1 (7.3, 9.0)	9385	− 0.1 (− 0.9, 0.8)	8185	0.1 (− 1.1, 1.2)
Non-response	182	2.0 (1.6, 2.3)	135	2.3 (1.8, 2.8)	137	2.4 (2.0, 3.0)	9385	0.2 (− 0.3, 0.7)	8185	0.3 (− 0.2, 0.7)
* Item score†*	*12,765*	*1.34 (1.32*,* 1.35)*	*9250*	*1.34 (1.33*,* 1.36)*	*8759*	*1.34 (1.33*,* 1.36)*	*9171*	*0.012 (− 0.006*,* 0.030)*	*8029*	*− 0.016 (− 0.037*,* 0.005)*
In the next few years, there will be civil war in the United States.										
Do not agree (1)	6407	47.6 (46.5, 48.8)	6167	63.2 (61.9, 64.6)	5768	62.2 (60.8, 63.6)	9385	15.1 (13.7, 16.5)	8185	− 1.7 (− 3.1, − 0.2)
Somewhat agree (2)	4746	36.7 (35.6, 37.7)	2576	28.3 (27.1, 29.6)	2524	28.8 (27.6, 30.2)	9385	− 8.0 (− 9.6, − 6.4)	8185	0.8 (− 0.7, 2.3)
Strongly or very strongly agree (3)	1604	13.7 (12.9, 14.5)	480	5.7 (5.1, 6.4)	465	6.5 (5.8, 7.4)	9385	− 7.7 (− 8.7, − 6.6)	8185	0.8 (− 0.1, 1.7)
Non-response	190	2.0 (1.7, 2.4)	162	2.7 (2.2, 3.2)	139	2.4 (1.9, 2.9)	9385	0.6 (0.0, 1.1)	8185	0.1 (− 0.4, 0.6)
* Item score†*	*12,757*	*1.65 (1.64*,* 1.67)*	*9223*	*1.41 (1.39*,* 1.43)*	*8757*	*1.43 (1.41*,* 1.45)*	*9149*	*− 0.236 (− 0.255*,* − 0.217)*	*8004*	***0.026 (0.007***,*** 0.045)***

Change scores have a potential range from 0 (no change) to ±2. Change scores for 2023-2034 that differ significantly from 0 are in bold font. Findings for 2022 and 2023 respondents and mean differences for 2022-2023 were published previously [10] and are reproduced for convenience

* Among respondents to both surveys

† Mean scores were calculated using values indicated in the response lines for individual items. Non-responses were excluded from mean score calculations and differences in mean scores were computed in the subsample of respondents with non-missing responses in both years by computing within-individual change scores and averaging them, to account for the longitudinal study design. For computing differences in individual response levels, indicator variables were computed for each item for each response level and within-individual differences in these were computed and averaged in the subsample of respondents who responded to the survey in both years. This explains the variation in the unweighted n for the mean differences

### American Society and Institutions

Four items explored beliefs on race and ethnicity (Table 4). There was a small decrease in agreement with the statement that “white people benefit from advantages in society that Black people do not have” (change − 0.043, 95% CI -0.062, -0.024) but also a small increase in agreement with the statement that “having more Black Americans, Latinos, and Asian Americans is good for the country” (change 0.028, 95% CI 0.007, 0.049). There was a small increase in agreement with the central element of QAnon mythology (change 0.026, 95% CI 0.008, 0.044) but no change for items regarding end-time Christianity (Table 5).

**Table 4:** Beliefs concerning race and ethnicity and American society

Statement	2022 Respondents (*n*= 12,947)		2023 Respondents (*n*=9385)		2024 Respondents (*n*=8896)		Mean Difference, * 2022-2023		Mean Difference, * 2023-2024	
	Unweighted *n*	Weighted % (95% CI) Mean score (95% CI)	Unweighted *n*	Weighted % (95% CI) Mean score (95% CI)	Unweighted *n*	Weighted % (95% CI) Mean score (95% CI)	Unweighted *n*	Weighted % (95% CI) Mean score (95% CI)	Unweighted *n*	Weighted % (95% CI) Mean score (95% CI)
White people benefit from advantages in society that Black people do not have.										
Do not agree (1)	4654	31.6 (30.6, 32.6)	3471	31.7 (30.5, 32.9)	3425	34.9 (33.5, 36.2)	9385	0.2 (− 0.9, 1.3)	8185	2.9 (1.7, 4.1)
Somewhat agree (2)	3665	27.8 (26.8, 28.8)	2828	29.9 (28.7, 31.2)	2712	29.1 (27.9, 30.4)	9385	2.5 (1.0, 3.9)	8185	− 0.9 (− 2.4, 0.7)
Strongly or very strongly agree (3)	4508	39.3 (38.2, 40.4)	2925	35.6 (34.2, 36.9)	2634	33.8 (32.5, 35.2)	9385	− 3.9 (− 5.1, − 2.8)	8185	− 1.6 (− 2.9, − 0.3)
Non-response	120	1.3 (1.1, 1.6)	161	2.8 (2.2, 3.3)	125	2.2 (1.7, 2.7)	9385	1.2 (0.7, 1.7)	8185	− 0.4 (− 1.0, 0.1)
* Item score†*	*12,827*	*2.08 (2.06*,* 2.10)*	*9224*	*2.04 (2.02*,* 2.06)*	*8771*	*1.99 (1.97*,* 2.01)*	*9181*	*− 0.042 (− 0.060*,* − 0.025)*	*7997*	***− 0.043 (− 0.062***,*** − 0.024)***
Discrimination against whites is as big a problem as discrimination against Blacks and other minorities.										
Do not agree (1)	6007	49.5 (48.4, 50.6)	4126	48.1 (46.7, 49.5)	3969	48.2 (46.8, 49.6)	9385	− 2.2 (− 3.4, − 0.9)	8185	0.3 (− 1.0, 1.6)
Somewhat agree (2)	3071	22.6 (21.7, 23.6)	2444	24.7 (23.6, 25.9)	2385	24.9 (23.8, 26.2)	9385	2.1 (0.7, 3.5)	8185	0.2 (− 1.3, 1.7)
Strongly or very strongly agree (3)	3759	26.6 (25.6, 27.6)	2682	24.9 (23.7, 26.0)	2415	24.7 (23.6, 26.0)	9385	− 0.9 (− 2.1, 0.3)	8185	− 0.4 (− 1.7, 0.8)
Non-response	110	1.2 (1.0, 1.5)	133	2.3 (1.8, 2.8)	127	2.1 (1.7, 2.6)	9385	0.9 (0.5, 1.4)	8185	0.0 (− 0.5, 0.5)
* Item score†*	*12,837*	*1.77 (1.75*,* 1.79)*	*9252*	*1.76 (1.74*,* 1.78)*	*8769*	*1.76 (1.74*,* 1.78)*	*9210*	*0.009 (− 0.011*,* 0.028)*	*8014*	*− 0.006 (− 0.026*,* 0.015)*
Having more Black Americans, Latinos, and Asian Americans is good for the country.										
Do not agree (1)	2774	18.5 (17.6, 19.3)	2323	20.7 (19.7, 21.8)	2168	21.3 (20.2, 22.4)	9385	2.9 (1.7, 4.0)	8185	− 0.6 (− 2.0, 0.9)
Somewhat agree (2)	4595	34.3 (33.2, 35.3)	3429	35.7 (34.4, 37.1)	3255	34.7 (33.4, 36.0)	9385	0.7 (− 0.9, 2.2)	8185	− 1.0 (− 2.4, 0.5)
Strongly or very strongly agree (3)	5320	45.1 (44.0, 46.2)	3338	39.6 (38.2, 40.9)	3204	40.1 (38.7, 41.5)	9385	− 5.0 (− 6.4, − 3.7)	8185	1.7 (0.6, 2.8)
Non-response	258	2.2 (1.9, 2.6)	295	4.0 (3.4, 4.5)	269	3.9 (3.3, 4.5)	9385	1.5 (0.9, 2.1)	8185	− 0.2 (− 0.8, 0.4)
* Item score†*	*12,689*	*2.27 (2.26*,* 2.29)*	*9090*	*2.20 (2.18*,* 2.22)*	*8627*	*2.20 (2.17*,* 2.22)*	*8979*	*− 0.079 (− 0.099*,* − 0.059)*	*7934*	***0.028 (0.007***,*** 0.049)***
In America, native-born white people are being replaced by immigrants.										
Do not agree (1)	7136	57.9 (56.8, 59.0)	5301	58.5 (57.1, 59.8)	4895	57.9 (56.5, 59.3)	9385	0.4 (− 1.1, 1.9)	8185	0.4 (− 0.8, 1.6)
Somewhat agree (2)	3483	25.0 (24.1, 26.0)	2099	21.7 (20.6, 22.9)	1941	20.8 (19.7, 22.0)	9385	− 3.0 (− 4.5, -1.5)	8185	− 1.6 (− 3.2, 0.1)
Strongly or very strongly agree (3)	2190	15.7 (14.9, 16.5)	1783	16.4 (15.5, 17.4)	1888	18.4 (17.4, 19.5)	9385	0.8 (− 0.3, 2.0)	8185	1.0 (− 0.5, 2.4)
Non-response	138	1.4 (1.1, 1.7)	202	3.4 (2.8, 3.9)	172	2.9 (2.4, 3.5)	9385	1.8 (1.2, 2.3)	8185	0.2 (− 0.4, 0.9)
* Item score†*	*12,809*	*1.57 (1.55*,* 1.59)*	*9183*	*1.57 (1.54*,* 1.59)*	*8724*	*1.59 (1.57*,* 1.62)*	*9121*	*− 0.007 (− 0.029*,* 0.016)*	*7806*	*0.010 (− 0.011*,* 0.031)*

Change scores have a potential range from 0 (no change) to ±2. Change scores for 2023-2034 that differ significantly from 0 are in bold font. Findings for 2022 and 2023 respondents and mean differences for 2022-2023 were published previously [10] and are reproduced for convenience

* Among respondents to both surveys

† Mean scores were calculated using values indicated in the response lines for individual items. Non-responses were excluded from mean score calculations and differences in mean scores were computed in the subsample of respondents with non-missing responses in both years by computing within-individual change scores and averaging them, to account for the longitudinal study design. For computing differences in individual response levels, indicator variables were computed for each item for each response level and within-individual differences in these were computed and averaged in the subsample of respondents who responded to the survey in both years. This explains the variation in the unweighted n for the mean differences

**Table 5:** Beliefs concerning QAnon and biblical “end times”

Statement	2022 Respondents (*n*= 12,947)		2023 Respondents (*n*=9385)		2024 Respondents (*n*=8896)		Mean Difference, * 2022-2023		Mean Difference, * 2023-2024	
	Unweighted *n*	Weighted % (95% CI) Mean score (95% CI)	Unweighted *n*	Weighted % (95% CI) Mean score (95% CI)	Unweighted *n*	Weighted % (95% CI) Mean score (95% CI)	Unweighted *n*	Weighted % (95% CI) Mean score (95% CI)	Unweighted *n*	Weighted % (95% CI) Mean score (95% CI)
The government, media, and financial worlds in the U.S. are controlled by a group of Satan-worshipping pedophiles who run a global child sex trafficking operation.										
Do not agree (1)	10,276	75.3 (74.2, 76.3)	7333	73.6 (72.3, 74.9)	6887	72.1 (70.7, 73.4)	9385	− 2.4 (− 3.5, -1.2)	8185	− 1.6 (− 2.9, − 0.4)
Somewhat agree (2)	1480	13.5 (12.7, 14.4)	1175	14.8 (13.7, 15.9)	1153	15.4 (14.3, 16.6)	9385	1.8 (0.6, 3.1)	8185	0.6 (− 0.8, 1.9)
Strongly or very strongly agree (3)	953	8.8 (8.1, 9.4)	681	8.7 (7.9, 9.6)	670	9.3 (8.5, 10.3)	9385	0.2 (− 0.7, 1.1)	8185	0.6 (− 0.4, 1.6)
Non-response	238	2.4 (2.1, 2.8)	196	2.9 (2.4, 3.4)	186	3.2 (2.6, 3.8)	9385	0.3 (− 0.2, 0.9)	8185	0.4 (− 0.1, 1.0)
* Item score†*	*12,709*	*1.32 (1.30*,* 1.33)*	*9189*	*1.33 (1.31*,* 1.35)*	*8710*	*1.35 (1.33*,* 1.37)*	*9088*	*0.025 (0.008*,* 0.042)*	*7935*	***0.026 (0.008***,*** 0.044)***
There is a storm coming soon that will sweep away the elites in power and restore the rightful leaders.										
Do not agree (1)	9064	68.1 (67.1, 69.2)	6735	68.9 (67.6, 70.3)	6316	68.3 (66.9, 69.6)	9385	0.6 (− 0.7, 2.0)	8185	− 1.0 (− 2.4, 0.4)
Somewhat agree (2)	2474	19.5 (18.6, 20.4)	1774	19.4 (18.3, 20.5)	1717	19.8 (18.7, 21.0)	9385	0.1 (− 1.2, 1.4)	8185	0.1 (− 1.3, 1.6)
Strongly or very strongly agree (3)	1162	9.8 (9.1, 10.5)	672	8.4 (7.6, 9.2)	669	8.6 (7.8, 9.5)	9385	− 1.3 (− 2.2, − 0.4)	8185	0.5 (− 0.5, 1.5)
Non-response	247	2.6 (2.2, 3.0)	204	3.3 (2.7, 3.8)	194	3.3 (2.8, 4.0)	9385	0.6 (0.0, 1.1)	8185	0.4 (− 0.2, 0.9)
* Item score†*	*12,700*	*1.40 (1.39*,* 1.42)*	*9181*	*1.37 (1.36*,* 1.39)*	*8702*	*1.38 (1.36*,* 1.40)*	*9075*	*− 0.020 (− 0.039*,* − 0.002)*	*7930*	*0.015 (− 0.005*,* 0.035)*
The chaos in America today is evidence that we are living in what the Bible calls “the end times.”										
Do not agree (1)	7412	54.7 (53.6, 55.8)	5536	56.4 (55.0, 57.7)	5423	57.6 (56.2, 59.0)	9385	0.8 (− 0.4, 2.0)	8185	0.3 (− 0.8, 1.5)
Somewhat agree (2)	3137	24.4 (23.4, 25.4)	2245	23.6 (22.4, 24.8)	2006	22.5 (21.3, 23.7)	9385	− 0.1 (− 1.4, 1.3)	8185	− 0.5 (− 1.9, 0.8)
Strongly or very strongly agree (3)	2225	19.0 (18.1, 19.9)	1453	17.5 (16.4, 18.6)	1332	17.5 (16.4, 18.7)	9385	− 1.4 (− 2.5, − 0.3)	8185	0.1 (− 1.0, 1.3)
Non-response	173	1.9 (1.5, 2.2)	151	2.6 (2.1, 3.1)	135	2.3 (1.9, 2.9)	9385	0.6 (0.1, 1.1)	8185	0.0 (− 0.4, 0.5)
* Item score†*	*12,774*	*1.64 (1.62*,* 1.65)*	*9234*	*1.60 (1.58*,* 1.62)*	*8761*	*1.59 (1.57*,* 1.61)*	*9159*	*− 0.020 (− 0.038*,* − 0.002)*	*8000*	*0.000 (− 0.020*,* 0.019)*

Change scores have a potential range from 0 (no change) to ±2. Change scores for 2023-2034 that differ significantly from 0 are in bold font. Findings for 2022 and 2023 respondents and mean differences for 2022-2023 were published previously [10] and are reproduced for convenience

* Among respondents to both surveys

† Mean scores were calculated using values indicated in the response lines for individual items. Non-responses were excluded from mean score calculations and differences in mean scores were computed in the subsample of respondents with non-missing responses in both years by computing within-individual change scores and averaging them, to account for the longitudinal study design. For computing differences in individual response levels, indicator variables were computed for each item for each response level and within-individual differences in these were computed and averaged in the subsample of respondents who responded to the survey in both years. This explains the variation in the unweighted n for the mean differences

### Political violence

There was no change from 2023 to 2024 in support for the uncommon view that political violence is usually or always justified “in general” or in the prevalence of the belief that violence was usually or always justified to advance at least 1 political objective (2024: 26.2%, 95% CI 25.0%, 27.5%; 2023: 25.3%, 95% CI 24.1%, 26.5%) (Table 6).

**Table 6:** Justification for political violence, in general and for 9 specific objectives

What do you think about the use of force or violence in the following situations?	2022 Respondents (*n*= 12,947)		2023 Respondents (*n*=9385)		2024 Respondents (*n*=8896)		Mean Difference, * 2022-2023		Mean Difference, * 2023-2024	
	Unweighted *n*	Weighted % (95% CI) Mean score (95% CI)	Unweighted *n*	Weighted % (95% CI) Mean score (95% CI)	Unweighted *n*	Weighted % (95% CI) Mean score (95% CI)	Unweighted *n*	Weighted % (95% CI) Mean score (95% CI)	Unweighted *n*	Weighted % (95% CI) Mean score (95% CI)
In general…to advance an important political objective that you support										
Never justified (1)	10,696	79.6 (78.6, 80.5)	7642	78.1 (76.9, 79.3)	7147	77.3 (76.0, 78.5)	9385	− 1.7 (− 3.0, − 0.5)	8185	− 0.9 (− 2.2, 0.4)
Sometimes justified (2)	1966	17.1 (16.2, 18.0)	1560	18.9 (17.7, 20.0)	1583	19.5 (18.4, 20.7)	9385	1.9 (0.7, 3.2)	8185	0.6 (− 0.6, 1.9)
Usually or always justified (3)	246	2.9 (2.5, 3.4)	136	2.2 (1.7, 2.7)	108	2.0 (1.6, 2.6)	9385	− 0.6 (− 1.3, 0.0)	8185	0.0 (− 0.7, 0.7)
Non-response	39	0.4 (0.2, 0.5)	47	0.8 (0.5, 1.1)	58	1.2 (0.8, 1.6)	9385	0.4 (0.2, 0.7)	8185	0.3 (− 0.1, 0.6)
* Item score†*	*12,908*	*1.23 (1.22*,* 1.24)*	*9338*	*1.23 (1.22*,* 1.25)*	*8838*	*1.24 (1.22*,* 1.25)*	*9325*	*0.009 (− 0.007*,* 0.025)*	*8109*	*0.008 (− 0.008*,* 0.024)*
Violence is usually or always justified to advance at least 1 of 17 objectives (unrestricted)‡	4386	32.5 (31.5, 33.6)	2684	28.2 (26.9, 29.4)	2834	30.0 (28.7, 31.3)	9385	− 3.9 (− 5.3, − 2.5)	8185	2.2 (0.8, 3.6)
Violence is usually or always justified to advance at least 1 of 17 objectives (restricted)‡	4386	32.5 (31.5, 33.6)	2361	25.3 (24.1, 26.5)	2432	26.2 (25.0, 27.5)	9385	− 6.8 (− 8.1, − 5.4)	8896	− 5.3 (− 6.8, − 3.9)
To return Donald Trump to the presidency this year§										
Never justified (1)	11,552	87.1 (86.3, 87.9)	8453	88.5 (87.5, 89.5)	7977	88.6 (87.6, 89.5)	9338	1.1 (0.1, 2.2)	8109	− 0.6 (− 1.7, 0.4)
Sometimes justified (2)	625	6.0 (5.4, 6.6)	375	4.9 (4.2, 5.6)	395	5.9 (5.1, 6.7)	9338	− 1.0 (− 1.9, − 0.1)	8109	1.1 (0.3, 2.0)
Usually or always justified (3)	616	5.3 (4.8, 5.8)	455	5.8 (5.1, 6.5)	400	4.6 (4.0, 5.2)	9338	0.3 (− 0.6, 1.1)	8109	− 0.8 (− 1.5, − 0.1)
Non-response	154	1.6 (1.3, 1.9)	55	0.8 (0.5, 1.1)	66	1.0 (0.7, 1.4)	9338	− 0.4 (− 0.8, − 0.1)	8109	0.3 (0.0, 0.6)
* Item score†*	*12,793*	*1.17 (1.16*,* 1.18)*	*9283*	*1.17 (1.15*,* 1.18)*	*8772*	*1.15 (1.14*,* 1.17)*	*9211*	*− 0.007 (− 0.023*,* 0.010)*	*8033*	*− 0.003 (− 0.019*,* 0.012)*
To stop an election from being stolen§										
Never justified (1)	9516	73.6 (72.6, 74.6)	7235	77.2 (76.0, 78.4)	6722	76.9 (75.7, 78.1)	9338	2.8 (1.5, 4.2)	8109	− 0.3 (− 1.7, 1.0)
Sometimes justified (2)	2219	16.7 (15.8, 17.5)	1388	14.8 (13.8, 15.8)	1433	15.4 (14.4, 16.4)	9338	− 1.7 (− 3.0, − 0.5)	8109	0.3 (− 1.0, 1.6)
Usually or always justified (3)	1065	8.3 (7.7, 8.9)	663	7.3 (6.5, 8.0)	624	6.8 (6.1, 7.6)	9338	− 0.8 (− 1.7, 0.1)	8109	− 0.2 (− 1.2, 0.7)
Non-response	147	1.5 (1.2, 1.8)	52	0.8 (0.5, 1.1)	59	0.9 (0.7, 1.3)	9338	− 0.3 (− 0.7, 0.1)	8109	0.3 (0.0, 0.6)
* Item score†*	*12,800*	*1.34 (1.32*,* 1.35)*	*9286*	*1.30 (1.28*,* 1.31)*	*8779*	*1.29 (1.28*,* 1.31)*	*9223*	*− 0.035 (− 0.054*,* − 0.016)*	*8044*	*0.000 (− 0.019*,* 0.020)*
To stop people who do not share my beliefs from voting§										
Never justified (1)	12,178	91.6 (90.9, 92.3)	8852	91.7 (90.8, 92.6)	8415	92.9 (92.0, 93.7)	9338	0.0 (− 0.9, 0.9)	8109	0.5 (− 0.5, 1.4)
Sometimes justified (2)	428	4.7 (4.1, 5.2)	277	4.8 (4.1, 5.6)	244	4.0 (3.3, 4.7)	9338	0.2 (− 0.6, 1.1)	8109	− 0.5 (− 1.4, 0.4)
Usually or always justified (3)	208	2.4 (2.0, 2.8)	159	2.7 (2.2, 3.2)	133	2.4 (1.9, 2.9)	9338	0.1 (− 0.5, 0.7)	8109	− 0.2 (− 0.8, 0.5)
Non-response	133	1.4 (1.1, 1.7)	50	0.8 (0.5, 1.0)	46	0.8 (0.5, 1.1)	9338	− 0.3 (− 0.7, 0.0)	8109	0.2 (− 0.1, 0.5)
* Item score†*	*12,814*	*1.10 (1.09*,* 1.11)*	*9288*	*1.10 (1.09*,* 1.12)*	*8792*	*1.09 (1.08*,* 1.10)*	*9227*	*0.004 (− 0.008*,* 0.017)*	*8050*	*− 0.006 (− 0.020*,* 0.007)*
To prevent discrimination based on race or ethnicity§										
Never justified (1)	8438	62.3 (61.2, 63.4)	6929	70.4 (69.1, 71.7)	6580	71.9 (70.6, 73.2)	9338	7.7 (6.1, 9.2)	8109	1.0 (− 0.5, 2.5)
Sometimes justified (2)	3388	27.1 (26.1, 28.1)	1750	20.3 (19.1, 21.4)	1691	20.4 (19.2, 21.6)	9338	− 6.7 (− 8.2, − 5.2)	8109	0.3 (− 1.1, 1.8)
Usually or always justified (3)	974	9.0 (8.3, 9.7)	607	8.5 (7.6, 9.4)	513	7.0 (6.2, 7.8)	9338	− 0.6 (− 1.6, 0.5)	8109	− 1.5 (− 2.5, − 0.5)
Non-response	147	1.5 (1.2, 1.8)	52	0.8 (0.5, 1.1)	54	0.8 (0.5, 1.1)	9338	− 0.4 (− 0.8, − 0.1)	8109	0.1 (− 0.2, 0.4)
* Item score†*	*12,800*	*1.46 (1.44*,* 1.47)*	*9286*	*1.38 (1.36*,* 1.39)*	*8784*	*1.35 (1.33*,* 1.36)*	*9216*	*− 0.081 (− 0.103*,* − 0.059)*	*8047*	***− 0.024 (− 0.046***,*** − 0.003)***
To preserve an American way of life based on Western European traditions§										
Never justified (1)	9329	74.2 (73.2, 75.1)	7267	79.2 (78.1, 80.3)	6723	78.1 (76.9, 79.2)	9338	4.8 (3.5, 6.1)	8109	− 1.5 (− 2.9, − 0.2)
Sometimes justified (2)	2705	18.6 (17.8, 19.5)	1513	14.4 (13.4, 15.3)	1608	16.2 (15.2, 17.3)	9338	− 4.1 (− 5.3, − 2.8)	8109	2.0 (0.6, 3.3)
Usually or always justified (3)	710	5.3 (4.8, 5.8)	483	5.5 (4.8, 6.2)	443	4.8 (4.2, 5.4)	9338	− 0.1 (− 0.9, 0.8)	8109	− 0.6 (− 1.5, 0.2)
Non-response	203	1.9 (1.6, 2.2)	75	1.0 (0.7, 1.3)	64	1.0 (0.7, 1.3)	9338	− 0.6 (− 1.0, − 0.2)	8109	0.2 (− 0.1, 0.6)
* Item score†*	*12,744*	*1.30 (1.29*,* 1.31)*	*9263*	*1.26 (1.24*,* 1.27)*	*8774*	*1.26 (1.24*,* 1.28)*	*9159*	*− 0.046 (− 0.064*,* − 0.029)*	*8023*	*0.008 (− 0.011*,* 0.027)*
To preserve an American way of life I believe in§										
Never justified (1)	6720	55.7 (54.6, 56.8)	6241	69.8 (68.6, 71.1)	5876	69.5 (68.2, 70.8)	9338	13.2 (11.7, 14.6)	8109	− 1.0 (− 2.5, 0.5)
Sometimes justified (2)	4449	31.6 (30.5, 32.6)	2221	20.9 (19.8, 22.1)	2188	21.8 (20.6, 22.9)	9338	− 10.7 (− 12.2, − 9.2)	8109	0.9 (− 0.6, 2.3)
Usually or always justified (3)	1697	11.9 (11.2, 12.6)	804	8.3 (7.5, 9.0)	714	7.8 (7.1, 8.6)	9338	− 2.8 (− 3.8, − 1.8)	8109	0.0 (− 1.0, 0.9)
Non-response	81	0.9 (0.6, 1.1)	72	1.0 (0.7, 1.3)	60	0.9 (0.6, 1.2)	9338	0.4 (0.0, 0.7)	8109	0.2 (− 0.1, 0.5)
* Item score†*	*12,866*	*1.56 (1.54*,* 1.57)*	*9266*	*1.38 (1.36*,* 1.40)*	*8778*	*1.38 (1.36*,* 1.40)*	*9236*	*− 0.164 (− 0.184*,* − 0.143)*	*8039*	*0.009 (− 0.011*,* 0.030)*
To oppose Americans who do not share my beliefs§										
Never justified (1)	11,746	88.5 (87.7, 89.3)	8564	88.7 (87.7, 89.7)	8135	89.7 (88.7, 90.6)	9338	− 0.2 (− 1.2, 0.9)	8109	0.3 (− 0.8, 1.4)
Sometimes justified (2)	871	7.9 (7.3, 8.6)	526	7.5 (6.7, 8.4)	487	6.9 (6.1, 7.7)	9338	− 0.5 (− 1.5, 0.5)	8109	− 0.2 (− 1.2, 0.9)
Usually or always justified (3)	263	2.8 (2.4, 3.2)	184	2.9 (2.4, 3.5)	170	2.8 (2.3, 3.4)	9338	0.2 (− 0.4, 0.9)	8109	− 0.2 (− 0.8, 0.5)
Non-response	67	0.7 (0.5, 1.0)	64	0.9 (0.6, 1.2)	46	0.7 (0.5, 1.0)	9338	0.4 (0.1, 0.7)	8109	0.0 (− 0.2, 0.3)
* Item score†*	*12,880*	*1.14 (1.13*,* 1.15)*	*9274*	*1.13 (1.12*,* 1.15)*	*8792*	*1.12 (1.11*,* 1.14)*	*9250*	*− 0.001 (− 0.015*,* 0.013)*	*8050*	*− 0.005 (− 0.019*,* 0.009)*
To oppose the government when it does not share my beliefs§										
Never justified (1)	10,607	80.2 (79.2, 81.1)	7890	82.2 (81.0, 83.3)	7459	82.3 (81.1, 83.5)	9338	2.1 (0.9, 3.3)	8109	− 0.5 (− 1.7, 0.8)
Sometimes justified (2)	1859	14.9 (14.1, 15.8)	1107	13.0 (12.0, 14.0)	1095	13.6 (12.6, 14.6)	9338	− 1.9 (− 3.1, − 0.7)	8109	0.7 (− 0.6, 2.0)
Usually or always justified (3)	338	3.4 (2.9, 3.8)	283	3.9 (3.3, 4.5)	230	3.3 (2.8, 4.0)	9338	0.2 (− 0.5, 0.9)	8109	− 0.3 (− 1.1, 0.5)
Non-response	143	1.5 (1.2, 1.8)	58	0.9 (0.6, 1.1)	54	0.8 (0.5, 1.1)	9338	− 0.4 (− 0.8, 0.0)	8109	0.1 (− 0.2, 0.4)
* Item score†*	*12,804*	*1.22 (1.21*,* 1.23)*	*9280*	*1.21 (1.20*,* 1.23)*	*8784*	*1.20 (1.19*,* 1.22)*	*9219*	*− 0.018 (− 0.034*,* − 0.001)*	*8039*	*0.002 (− 0.015*,* 0.018)*
To oppose the government when it tries to take private land for public purposes§										
Never justified (1)	7870	60.7 (59.6, 61.8)	6336	67.6 (66.2, 68.9)	5795	65.7 (64.3, 67.1)	9338	6.3 (4.8, 7.8)	8109	− 1.8 (− 3.3, − 0.3)
Sometimes justified (2)	3787	28.3 (27.3, 29.3)	2260	23.4 (22.2, 24.5)	2295	24.9 (23.7, 26.2)	9338	− 4.4 (− 6.0, − 2.9)	8109	1.5 (− 0.1, 3.0)
Usually or always justified (3)	1141	9.5 (8.8, 10.2)	682	8.2 (7.4, 9.0)	693	8.5 (7.7, 9.4)	9338	− 1.5 (− 2.5, − 0.5)	8109	0.3 (− 0.7, 1.3)
Non-response	149	1.5 (1.2, 1.8)	60	0.9 (0.6, 1.2)	55	0.8 (0.6, 1.2)	9338	− 0.3 (− 0.7, 0.1)	8109	0.1 (− 0.2, 0.4)
* Item score†*	*12,798*	*1.48 (1.47*,* 1.50)*	*9278*	*1.40 (1.38*,* 1.42)*	*8783*	*1.42 (1.40*,* 1.44)*	*9204*	*− 0.078 (− 0.098*,* − 0.058)*	*8034*	***0.022 (0.001***,*** 0.042)***

Change scores have a potential range from 0 (no change) to ±2. Change scores for 2023-2034 that differ significantly from 0 are in bold font. Findings for 2022 and 2023 respondents and mean differences for 2022-2023 were published previously [10] and are reproduced for convenience

* Among respondents to both surveys

† Mean scores were calculated using values indicated in the response lines for individual items. Non-responses were excluded from mean score calculations and differences in mean scores were computed in the subsample of respondents with non-missing responses in both years by computing within-individual change scores and averaging them, to account for the longitudinal study design. For computing differences in individual response levels, indicator variables were computed for each item for each response level and within-individual differences in these were computed and averaged in the subsample of respondents who responded to the survey in both years. This explains the variation in the unweighted n for the mean differences

‡ In the unrestricted version, the computation for each respondent is based on all objectives presented to that respondent in that year. In the restricted version, the computation for each respondent is based on the 13 objectives presented to that respondent in all 3 years

§ In each year, participants who did not answer the question “In general…to advance an important political objective that you support” were not asked this question

Among 17 objectives considered individually (Tables 6, 7), there was a small increase in the belief that violence was justified in 5 cases: “to oppose the government when it tries to take private land for public purposes” (change 0.022, 95% CI 0.001, 0.042), “to stop police violence” (change 0.027, 95% CI 0.005, 0.050), “to reinforce the police” (change 0.025, 95% CI 0.003, 0.046), “to stop illegal immigration” (change 0.046, 95% CI 0.026, 0.067), and “to stop a protest” (change 0.046, 95% CI 0.027, 0.065). There was a small decrease in the belief that violence was justified “To prevent discrimination based on race or ethnicity” (change − 0.024, 95% CI -0.046, -0.003).

**Table 7:** Justification for political violence for 8 additional specific objectives*

What do you think about the use of force or violence in the following situations?	2022 Respondents (*n*= 12,947)		2023 Respondents (*n*=9385)		2024 Respondents (*n*=8896)		Mean Difference†, 2022-2023		Mean Difference†, 2023-2024	
	Unweighted *n*	Weighted % (95% CI) Mean score (95% CI)	Unweighted *n*	Weighted % (95% CI) Mean score (95% CI)	Unweighted *n*	Weighted % (95% CI) Mean score (95% CI)	Unweighted *n*	Weighted % (95% CI) Mean score (95% CI)	Unweighted *n*	Weighted % (95% CI) Mean score (95% CI)
To stop voter fraud‡										
Never justified (1)	4772	73.3 (71.9, 74.7)	7180	77.2 (76.0, 78.4)	6762	77.2 (76.0, 78.4)	4697	3.1 (1.2, 4.9)	8109	− 0.3 (− 1.7, 1.1)
Sometimes justified (2)	1023	16.3 (15.2, 17.5)	1292	13.4 (12.4, 14.4)	1328	14.4 (13.4, 15.4)	4697	− 2.5 (− 4.4, − 0.6)	8109	1.1 (− 0.2, 2.4)
Usually or always justified (3)	624	9.4 (8.5, 10.4)	798	8.5 (7.8, 9.4)	703	7.7 (7.0, 8.5)	4697	− 0.7 (− 2.1, 0.7)	8109	− 0.8 (− 1.7, 0.2)
Non-response	43	1.0 (0.7, 1.4)	68	0.9 (0.7, 1.2)	45	0.7 (0.5, 1.0)	4697	− 0.1(− 0.3, 0.5)	8109	0.0 (− 0.3, 0.2)
* Item score§*	*6419*	*1.35 (1.33*,* 1.38)*	*9270*	*1.31 (1.29*,* 1.32)*	*8793*	*1.30 (1.28*,* 1.32)*	*4650*	*− 0.038 (− 0.065*,* − 0.011)*	*8044*	*− 0.004 (− 0.024*,* 0.015)*
To stop voter intimidation‡										
Never justified (1)	3847	61.2 (59.7, 62.7)	6478	70.4 (69.1, 71.7)	5997	68.9 (67.5, 70.2)	4641	8.4 (6.2, 10.6)	8109	− 2.1 (− 3.6, − 0.6)
Sometimes justified (2)	1903	27.9 (26.5, 29.3)	2050	20.8 (19.7, 22.0)	2129	22.9 (21.7, 24.1)	4641	− 7.1 (− 9.2, − 4.9)	8109	2.5 (1.1, 4.0)
Usually or always justified (3)	705	10.3 (9.3, 11.3)	742	7.8 (7.1, 8.6)	658	7.5 (6.7, 8.3)	4641	− 2.1 (− 3.6, − 0.6)	8109	− 0.4 (− 1.4, 0.5)
Non-response	30	0.6 (0.4, 1.0)	68	0.9 (0.7, 1.3)	54	0.8 (0.5, 1.1)	4641	0.8 (0.3, 1.2)	8109	0.0 (− 0.2, 0.3)
* Item score§*	*6455*	*1.49 (1.47*,* 1.51)*	*9270*	*1.37 (1.35*,* 1.39)*	*8784*	*1.38 (1.36*,* 1.40)*	*4597*	*− 0.113 (− 0.143*,* − 0.082)*	*8039*	*0.018 (− 0.003*,* 0.038)*
To stop police violence‡										
Never justified (1)	3114	45.5 (43.9, 47.1)	5493	57.7 (56.3, 59.0)	4934	55.3 (53.9, 56.8)	4666	10.9 (8.6, 13.2)	8109	− 2.2 (− 3.9, − 0.6)
Sometimes justified (2)	2580	41.0 (39.5, 42.6)	2970	31.5 (30.2, 32.8)	3064	33.8 (32.4, 35.1)	4666	− 8.2 (− 10.6, − 5.8)	8109	1.8 (0.2, 3.5)
Usually or always justified (3)	731	12.7 (11.7, 13.9)	807	9.9 (9.1, 10.9)	786	10.1 (9.2, 11.1)	4666	− 3.0 (− 4.5, − 1.5)	8109	0.4 (− 0.8, 1.5)
Non-response	37	0.8 (0.5, 1.1)	68	0.9 (0.7, 1.3)	54	0.8 (0.5, 1.1)	4666	0.3 (0.0, 0.6)	8109	0.0 (− 0.2, 0.3)
* Item score§*	*6425*	*1.67 (1.65*,* 1.69)*	*9270*	*1.52 (1.50*,* 1.54)*	*8784*	*1.54 (1.52*,* 1.56)*	*4619*	*− 0.141 (− 0.171*,* − 0.111)*	*8042*	***0.027 (0.005***,*** 0.050)***
To reinforce the police‡										
Never justified (1)	2377	42.2 (40.6, 43.8)	4851	58.2 (56.9, 59.6)	4346	56.5 (55.1, 57.9)	4672	14.9 (12.7, 17.1)	8109	− 1.9 (− 3.5, − 0.3)
Sometimes justified (2)	2661	38.7 (37.2, 40.2)	3279	29.8 (28.6, 31.1)	3238	31.5 (30.2, 32.8)	4672	− 7.8 (− 10.1, − 5.5)	8109	1.3 (− 0.3, 2.9)
Usually or always justified (3)	1404	18.3 (17.2, 19.5)	1139	11.0 (10.2, 11.9)	1200	11.3 (10.4, 12.1)	4672	− 7.8 (− 9.4, − 6.2)	8109	0.5 (− 0.6, 1.5)
Non-response	43	0.9 (0.6, 1.2)	69	0.9 (0.7, 1.3)	54	0.8 (0.5, 1.1)	4672	0.7 (0.2, 1.2)	8109	0.1 (− 0.2, 0.4)
* Item score§*	*6442*	*1.76 (1.74*,* 1.78)*	*9269*	*1.52 (1.50*,* 1.54)*	*8784*	*1.54 (1.53*,* 1.56)*	*4619*	*− 0.231 (− 0.262*,* − 0.200)*	*8039*	***0.025 (0.003***,*** 0.046)***
To stop illegal immigration‡										
Never justified (1)	3733	61.0 (59.4, 62.5)	5757	65.7 (64.3, 66.9)	5204	63.3 (62.0, 64.7)	4658	3.7 (1.7, 5.7)	8109	− 2.5 (− 3.9, − 1.0)
Sometimes justified (2)	1819	26.5 (25.1, 27.9)	2341	22.3 (21.2, 23.5)	2249	22.8 (21.7, 24.0)	4658	− 5.1 (− 7.1, − 3.0)	8109	0.2 (− 1.3, 1.6)
Usually or always justified (3)	858	11.5 (10.6, 12.6)	1174	11.1 (10.3, 12.0)	1339	13.0 (12.1, 14.0)	4658	1.1 (− 0.5, 2.6)	8109	2.2 (1.2, 3.3)
Non-response	39	1.0 (0.7, 1.5)	66	0.9 (0.7, 1.3)	46	0.8 (0.6, 1.2)	4658	0.3 (− 0.1, 0.6)	8109	0.1 (− 0.2, 0.4)
* Item score§*	*6410*	*1.50 (1.48*,* 1.52)*	*9272*	*1.45 (1.43*,* 1.47)*	*8792*	*1.49 (1.47*,* 1.51)*	*4615*	*− 0.028 (− 0.057*,* 0.002)*	*8044*	***0.046 (0.026***,*** 0.067)***
To keep borders open‡										
Never justified (1)	4401	66.2 (64.7, 67.7)	7477	78.0 (76.8, 79.2)	7107	79.1 (77.9, 80.3)	4658	11.4 (9.3, 13.5)	8109	0.7 (− 0.7, 2.1)
Sometimes justified (2)	1535	24.9 (23.5, 26.3)	1295	14.9 (13.9, 15.9)	1187	14.0 (13.0, 15.0)	4658	− 9.1 (− 11.2, − 7.0)	8109	− 1.1 (− 2.4, 0.2)
Usually or always justified (3)	518	8.2 (7.3, 9.1)	495	6.1 (5.5, 6.9)	491	5.9 (5.3, 6.7)	4658	− 2.6 (− 3.9, − 1.3)	8109	0.2 (− 0.7, 1.1)
Non-response	44	0.7 (0.5, 1.0)	71	0.9 (0.7, 1.3)	53	1.0 (0.7, 1.4)	4658	0.3 (− 0.1, 0.8)	8109	0.2 (− 0.2, 0.5)
* Item score§*	*6454*	*1.42 (1.39*,* 1.44)*	*9267*	*1.27 (1.26*,* 1.29)*	*8785*	*1.26 (1.24*,* 1.28)*	*4624*	*− 0.143 (− 0.171*,* − 0.115)*	*8040*	*− 0.006 (− 0.025*,* 0.013)*
To stop a protest‡										
Never justified (1)	3682	57.8 (56.2, 59.3)	6599	72.0 (70.7, 73.2)	5749	67.1 (65.7, 68.4)	4656	12.8 (10.7, 15.0)	8109	− 4.4 (− 5.9, − 2.9)
Sometimes justified (2)	2396	35.3 (33.8, 36.8)	2233	21.4 (20.4, 22.6)	2557	26.5 (25.3, 27.8)	4656	− 12.6 (− 14.8, − 10.4)	8109	4.2 (2.7, 5.7)
Usually or always justified (3)	376	6.0 (5.3, 6.9)	434	5.6 (5.0, 6.4)	485	5.7 (5.1, 6.4)	4656	− 0.6 (− 1.7, 0.5)	8109	0.2 (− 0.6, 1.1)
Non-response	41	0.9 (0.6, 1.3)	72	1.0 (0.7, 1.3)	47	0.7 (0.5, 1.0)	4656	0.4 (0.0, 0.8)	8109	0.0 (− 0.3, 0.3)
* Item score§*	*6454*	*1.48 (1.46*,* 1.50)*	*9266*	*1.33 (1.31*,* 1.35)*	*8791*	*1.38 (1.36*,* 1.40)*	*4608*	*− 0.137 (− 0.164*,* − 0.110)*	*8039*	***0.046 (0.027***,*** 0.065)***
To support a protest‡										
Never justified (1)	5244	78.4 (77.1, 79.7)	7783	80.5 (79.3, 81.6)	7389	80.7 (79.4, 81.8)	4682	2.1 (0.2, 4.1)	8109	− 0.1 (− 1.4, 1.3)
Sometimes justified (2)	935	16.4 (15.2, 17.7)	1174	14.1 (13.2, 15.2)	1142	14.9 (13.9, 16.1)	4682	− 2.6 (− 4.4, − 0.8)	8109	0.3 (− 1.1, 1.7)
Usually or always justified (3)	246	4.5 (3.9, 5.2)	319	4.5 (3.9, 5.2)	260	3.7 (3.1, 4.3)	4682	0.0 (− 1.2, 1.1)	8109	− 0.4 (− 1.2, 0.4)
Non-response	27	0.6 (0.4, 1.0)	62	0.9 (0.6, 1.2)	47	0.7 (0.5, 1.1)	4682	0.5 (0.1, 1.0)	8109	0.1 (− 0.2, 0.4)
* Item score§*	*6425*	*1.26 (1.24*,* 1.27)*	*9276*	*1.23 (1.22*,* 1.25)*	*8791*	*1.22 (1.21*,* 1.24)*	*4641*	*− 0.027 (− 0.053*,* − 0.002)*	*8044*	*− 0.003 (− 0.021*,* 0.015)*

Change scores have a potential range from 0 (no change) to ±2. Change scores for 2023-2034 that differ significantly from 0 are in bold font. Findings for 2022 and 2023 respondents and mean differences for 2022-2023 were published previously [10] and are reproduced for convenience

* These objectives were paired in 2022, with respondents randomized 1:1 to see 1 item in each pair

† Among respondents to both surveys

‡ Participants in each year who did not answer the question “In general…to advance an important political objective that you support” were not asked this question

§ Mean scores were calculated using values indicated in the response lines for individual items. Non-responses were excluded from mean score calculations and differences in mean scores were computed in the subsample of respondents with non-missing responses in both years by computing within-individual change scores and averaging them, to account for the longitudinal study design. For computing differences in individual response levels, indicator variables were computed for each item for each response level and within-individual differences in these were computed and averaged in the subsample of respondents who responded to the survey in both years. This explains the variation in the unweighted n for the mean differences

**Fig. 1:**
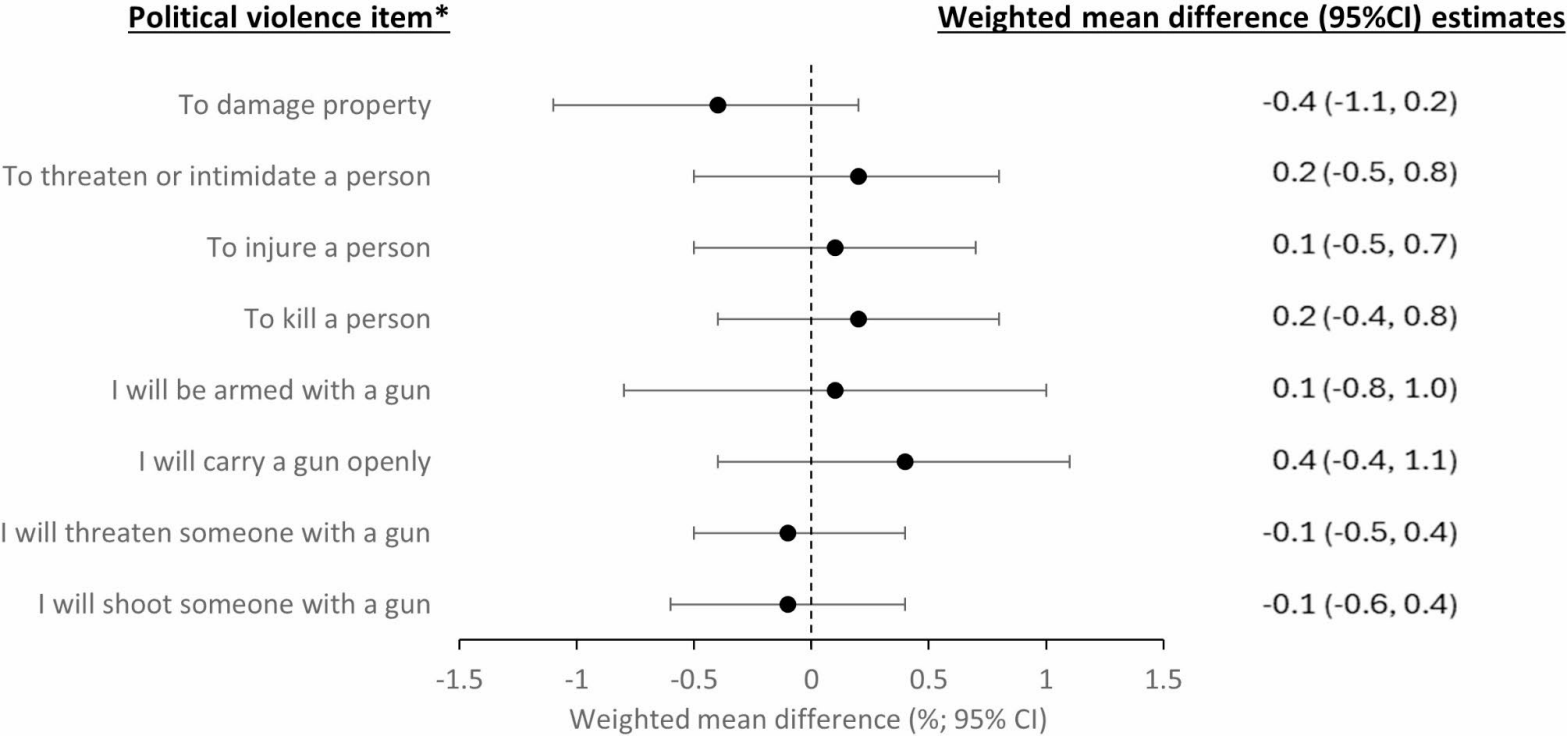
Difference in willingness to engage in political violence and expectation of firearm use. *Items 1–4: Personal willingness to use violence to achieve a political objective (very or completely willing). Items 5–8: Likelihood of using a gun in the future to achieve a political objective (very or extremely likely)

The proportion of respondents who were not asked questions about their personal willingness to use force or violence to advance a political objective decreased slightly from 2023 to 2024 (− 2.9%, 95% CI -4.4%, − 1.3%) (Table 8). This reflects the small increase in respondents who considered political violence to be at least sometimes justified for at least 1 of the 17 specified objectives. Among those asked, there was no overall change in willingness to “damage property,” “threaten or intimidate a person,” “injure a person,” or “kill a person” (Table 8, Fig. 1).

**Table 8:** Personal willingness to engage in political violence, by type of violence

In a situation where you think force or violence is justified to advance an important political objective…How willing would you personally be to use force or violence in each of these ways?	2022 Respondents (*n*= 12,947)		2023 Respondents (*n*=9385)		2024 Respondents (*n*=8896)		Mean Difference, * 2022-2023		Mean Difference, * 2023-2024	
	Unweighted *n*	Weighted % (95% CI) Mean score (95% CI)	Unweighted *n*	Weighted % (95% CI) Mean score (95% CI)	Unweighted *n*	Weighted % (95% CI) Mean score (95% CI)	Unweighted *n*	Weighted % (95% CI) Mean score (95% CI)	Unweighted *n*	Weighted % (95% CI) Mean score (95% CI)
To damage property										
Not asked the question†	2558	21.8 (20.9, 22.8)	2468	30.2 (28.9, 31.5)	2013	27.0 (25.8, 28.4)	9338	7.8 (6.3, 9.2)	8109	− 2.9 (− 4.4, − 1.3)
Not willing (1)	9101	66.9 (65.8, 68.0)	5856	57.3 (55.9, 58.7)	5978	61.5 (60.1, 62.9)	9338	− 9.2 (− 10.8, − 7.5)	8109	3.5 (1.7, 5.2)
Somewhat willing (2)	920	7.6 (7.0, 8.2)	755	9.0 (8.2, 9.8)	619	8.2 (7.4, 9.1)	9338	1.3 (0.3, 2.4)	8109	− 0.3 (− 1.4, 0.7)
Very or completely willing (3)	303	2.9 (2.5, 3.4)	224	3.0 (2.5, 3.6)	188	2.6 (2.2, 3.2)	9338	0.1 (− 0.5, 0.7)	8109	− 0.4 (− 1.1, 0.2)
Non-response	65	0.7 (0.5, 1.0)	35	0.5 (0.4, 0.8)	40	0.6 (0.4, 0.9)	9338	− 0.1 (− 0.4, 0.2)	8109	0.1 (− 0.2, 0.4)
* Item score‡*	*10,324*	*1.17 (1.16*,* 1.19)*	*6835*	*1.22 (1.20*,* 1.24)*	*6785*	*1.19 (1.17*,* 1.20)*	*5960*	*0.033 (0.011*,* 0.054)*	*5158*	*− 0.018 (− 0.039*,* 0.004)*
To threaten or intimidate a person										
Not asked the question†	2558	21.8 (20.9, 22.8)	2468	30.2 (28.9, 31.5)	2013	27.0 (25.8, 28.4)	9338	7.8 (6.3, 9.2)	8109	− 2.9 (− 4.4, − 1.3)
Not willing (1)	9221	67.8 (66.8, 68.9)	5900	58.5 (57.1, 59.8)	5954	62.1 (60.7, 63.5)	9338	− 8.8 (− 10.5, − 7.2)	8109	2.9 (1.1, 4.7)
Somewhat willing (2)	883	7.5 (6.8, 8.1)	746	8.3 (7.5, 9.1)	663	7.8 (7.0, 8.6)	9338	0.8 (− 0.1, 1.7)	8109	− 0.3 (− 1.4, 0.8)
Very or completely willing (3)	210	2.0 (1.7, 2.4)	177	2.4 (2.0, 3.0)	161	2.4 (1.9, 2.9)	9338	0.3 (− 0.2, 0.9)	8109	0.2 (− 0.5, 0.8)
Non-response	75	0.8 (0.6, 1.1)	47	0.6 (0.5, 0.9)	47	0.7 (0.5, 1.0)	9338	− 0.1 (− 0.4, 0.2)	8109	0.1 (− 0.2, 0.4)
* Item score‡*	*10,314*	*1.15 (1.14*,* 1.16)*	*6823*	*1.19 (1.17*,* 1.21)*	*6778*	*1.17 (1.16*,* 1.19)*	*5942*	*0.025 (0.005*,* 0.046)*	*5144*	*− 0.001 (− 0.024*,* 0.021)*
To injure a person										
Not asked the question†	2558	21.8 (20.9, 22.8)	2468	30.2 (28.9, 31.5)	2013	27.0 (25.8, 28.4)	9338	7.8 (6.3, 9.2)	8109	− 2.9 (− 4.4, − 1.3)
Not willing (1)	9374	69.3 (68.3, 70.4)	6137	60.6 (59.2, 62.0)	6146	64.2 (62.8, 65.6)	9338	− 8.4 (− 10.1, − 6.8)	8109	3.0 (1.2, 4.7)
Somewhat willing (2)	709	6.0 (5.4, 6.6)	521	6.2 (5.5, 7.0)	474	5.9 (5.2, 6.6)	9338	0.6 (− 0.3, 1.4)	8109	− 0.2 (− 1.1, 0.8)
Very or completely willing (3)	217	2.0 (1.7, 2.4)	158	2.2 (1.8, 2.7)	155	2.2 (1.8, 2.7)	9338	0.0 (− 0.5, 0.6)	8109	0.1 (− 0.5, 0.7)
Non-response	89	0.9 (0.7, 1.1)	54	0.8 (0.6, 1.1)	50	0.7 (0.5, 1.0)	9338	0.0 (− 0.3, 0.4)	8109	0.0 (− 0.4, 0.3)
* Item score‡*	*10,300*	*1.13 (1.12*,* 1.14)*	*6816*	*1.15 (1.14*,* 1.17)*	*6775*	*1.14 (1.13*,* 1.16)*	*5931*	*0.016 (− 0.005*,* 0.036)*	*5142*	*0.000 (− 0.022*,* 0.021)*
To kill a person										
Not asked the question†	2558	21.8 (20.9, 22.8)	2468	30.2 (28.9, 31.5)	2013	27.0 (25.8, 28.4)	9338	7.8 (6.3, 9.2)	8109	− 2.9 (− 4.4, − 1.3)
Not willing (1)	9666	71.9 (70.9, 73.0)	6388	63.6 (62.2, 65.0)	6317	66.4 (65.0, 67.7)	9338	− 7.8 (− 9.4, − 6.2)	8109	2.2 (0.5, 3.9)
Somewhat willing (2)	423	3.4 (3.0, 3.9)	292	3.7 (3.1, 4.3)	318	3.8 (3.3, 4.5)	9338	0.3 (− 0.4, 1.0)	8109	0.3 (− 0.5, 1.1)
Very or completely willing (3)	225	1.9 (1.6, 2.3)	142	1.8 (1.5, 2.3)	136	2.0 (1.6, 2.5)	9338	− 0.2 (− 0.7, 0.4)	8109	0.2 (− 0.4, 0.8)
Non-response	75	0.8 (0.6, 1.1)	48	0.7 (0.5, 1.0)	54	0.8 (0.6, 1.1)	9338	− 0.1 (− 0.5, 0.3)	8109	0.2 (− 0.2, 0.6)
* Item score‡*	*10,314*	*1.09 (1.08*,* 1.10)*	*6870*	*1.09 (1.07*,* 1.10)*	*6771*	*1.11 (1.09*,* 1.12)*	*5943*	*0.004 (− 0.015*,* 0.022)*	5136	*0.016 (− 0.003*,* 0.036)*

Change scores have a potential range from 0 (no change) to ±2. Change scores for 2023-2034 that differ significantly from 0 are in bold font. Findings for 2022 and 2023 respondents and mean differences for 2022-2023 were published previously [10] and are reproduced for convenience

* Among respondents to both surveys

† These respondents answered “never justified” to all prior questions on the use of force or violence to advance specific political objectives. They were not asked questions on their personal willingness to use political violence

‡ Mean scores were calculated using values indicated in the response lines for individual items. Non-responses were excluded from mean score calculations and differences in mean scores were computed in the subsample of respondents with non-missing responses in both years by computing within-individual change scores and averaging them, to account for the longitudinal study design. For computing differences in individual response levels, indicator variables were computed for each item for each response level and within-individual differences in these were computed and averaged in the subsample of respondents who responded to the survey in both years. This explains the variation in the unweighted n for the mean differences

There were small increases in willingness to use force or violence to advance a political objective against 3 groups of people (Table 9): “a person who does not share your race or ethnicity” (change 0.048, 95% CI 0.029, 0.067), “a person who does not share your religion” (change 0.051, 95% CI 0.032, 0.070), and “a person who does not share your political beliefs” (change 0.042, 95% CI 0.023, 0.062).

**Table 9:** Personal willingness to engage in political violence, by target of violence

In a situation where you think force or violence is justified to advance an important political objective…How willing would you personally be to use force or violence in each of these ways?	2022 Respondents (*n*= 12,947)		2023 Respondents (*n*=9385)		2024 Respondents (*n*=8896)		Mean Difference, * 2022-2023		Mean Difference, * 2023-2024	
	Unweighted *n*	Weighted % (95% CI) Mean score (95% CI)	Unweighted *n*	Weighted % (95% CI) Mean score (95% CI)	Unweighted *n*	Weighted % (95% CI) Mean score (95% CI)	Unweighted *n*	Weighted % (95% CI) Mean score (95% CI)	Unweighted *n*	Weighted % (95% CI) Mean score (95% CI)
An elected federal or state government official										
Not asked the question†	2558	21.8 (20.9, 22.8)	2468	30.2 (28.9, 31.5)	2013	27.0 (25.8, 28.4)	9338	7.8 (6.3, 9.2)	8109	− 2.9 (− 4.4, − 1.3)
Not willing	9509	70.5 (69.5, 71.5)	6301	62.0 (60.6, 63.4)	6266	65.4 (64.0, 66.8)	9338	− 8.6 (− 10.2, − 7.0)	8109	3.6 (1.9, 5.4)
Somewhat willing	582	4.6 (4.1, 5.1)	359	4.8 (4.2, 5.6)	356	4.7 (4.1, 5.4)	9338	0.5 (− 0.4, 1.3)	8109	− 0.5 (− 1.5, 0.6)
Very or completely willing	186	1.9 (1.6, 2.3)	129	1.8 (1.4, 2.2)	143	2.0 (1.6, 2.5)	9338	0.1 (− 0.4, 0.7)	8109	− 0.4 (− 1.1, 0.2)
Non-response	112	1.1 (0.9, 1.4)	81	1.2 (0.9, 1.6)	60	0.8 (0.6, 1.1)	9338	0.2 (− 0.3, 0.6)	8109	0.1 (− 0.2, 0.5)
* Item score‡*	*10,277*	*1.11 (1.10*,* 1.12)*	*6789*	*1.12 (1.11*,* 1.14)*	*6765*	*1.12 (1.11*,* 1.14)*	*5900*	*− 0.014 (− 0.032*,* 0.005)*	*5158*	*− 0.020 (− 0.042*,* 0.001)*
An elected local government official										
Not asked the question†	2558	21.8 (20.9, 22.8)	2468	30.2 (28.9, 31.5)	2013	27.0 (25.8, 28.4)	9338	7.8 (6.3, 9.2)	8109	− 2.9 (− 4.4, − 1.3)
Not willing	9582	71.1 (70.1, 72.1)	6347	62.7 (61.4, 64.1)	6302	66.0 (64.6, 67.3)	9338	− 7.8 (− 9.4, − 6.2)	8109	3.0 (1.2, 4.8)
Somewhat willing	515	4.2 (3.7, 4.7)	327	4.2 (3.6, 4.9)	333	4.3 (3.7, 5.0)	9338	− 0.2 (− 1.0, 0.6)	8109	− 0.5 (− 1.6, 0.7)
Very or completely willing	168	1.6 (1.3, 2.0)	118	1.8 (1.4, 2.2)	127	1.9 (1.5, 2.4)	9338	0.1 (− 0.3, 0.6)	8109	0.2 (− 0.4, 0.7)
Non-response	124	1.2 (1.0, 1.5)	78	1.1 (0.9, 1.5)	63	0.8 (0.6, 1.1)	9338	0.1 (− 0.3, 0.5)	8109	0.1 (− 0.2, 0.5)
* Item score‡*	*10,265*	*1.10 (1.09*,* 1.11)*	*6792*	*1.11 (1.10*,* 1.13)*	*6762*	*1.11 (1.10*,* 1.13)*	*5898*	*0.000 (− 0.017*,* 0.017)*	*5144*	*− 0.003 (− 0.025*,* 0.019)*
An election worker, such as a poll worker or vote counter										
Not asked the question†	2558	21.8 (20.9, 22.8)	2468	30.2 (28.9, 31.5)	2013	27.0 (25.8, 28.4)	9338	7.8 (6.3, 9.2)	8109	− 2.9 (− 4.4, − 1.3)
Not willing	9874	72.9 (71.9, 73.9)	6507	64.1 (62.7, 65.5)	6472	67.7 (66.3, 69.0)	9338	− 8.3 (− 9.8, − 6.7)	8109	3.0 (1.3, 4.8)
Somewhat willing	283	2.7 (2.3, 3.1)	186	3.0 (2.5, 3.6)	184	2.5 (2.1, 3.1)	9338	0.3 (− 0.4, 1.0)	8109	− 0.3 (− 1.2, 0.7)
Very or completely willing	131	1.5 (1.2, 1.8)	104	1.6 (1.3, 2.1)	107	1.9 (1.4, 2.4)	9338	0.0 (− 0.5, 0.5)	8109	0.1 (− 0.5, 0.7)
Non-response	101	1.1 (0.9, 1.4)	73	1.1 (0.8, 1.5)	62	0.9 (0.7, 1.2)	9338	0.2 (− 0.2, 0.5)	8109	0.0 (− 0.4, 0.3)
* Item score‡*	*10,288*	*1.07 (1.06*,* 1.08)*	*6797*	*1.09 (1.08*,* 1.11)*	*6763*	*1.09 (1.07*,* 1.10)*	*5915*	*0.010 (− 0.006*,* 0.026)*	*5142*	*− 0.001 (− 0.022*,* 0.020)*
A public health official										
Not asked the question†	2558	21.8 (20.9, 22.8)	2468	30.2 (28.9, 31.5)	2013	27.0 (25.8, 28.4)	9338	7.8 (6.3, 9.2)	8109	− 2.9 (− 4.4, − 1.3)
Not willing	9750	72.1 (71.0, 73.1)	6433	63.6 (62.2, 64.9)	6398	66.8 (65.4, 68.2)	9338	− 7.8 (− 9.4, − 6.3)	8109	2.2 (0.5, 3.9)
Somewhat willing	386	3.4 (3.0, 3.8)	233	3.3 (2.8, 4.0)	237	3.2 (2.7, 3.9)	9338	− 0.1 (− 0.9, 0.6)	8109	0.2 (− 0.6, 1.0)
Very or completely willing	137	1.5 (1.2, 1.9)	126	1.9 (1.5, 2.3)	117	1.9 (1.5, 2.5)	9338	0.1 (− 0.4, 0.7)	8109	0.3 (− 0.3, 0.9)
Non-response	116	1.2 (1.0, 1.5)	78	1.1 (0.8, 1.5)	73	1.0 (0.7, 1.3)	9338	0.1 (− 0.4, 0.5)	8109	0.2 (− 0.2, 0.5)
* Item score‡*	*10,273*	*1.08 (1.07*,* 1.09)*	*6792*	*1.10 (1.09*,* 1.12)*	*6752*	*1.10 (1.08*,* 1.11)*	*5904*	*0.001 (− 0.015*,* 0.018)*	*5136*	*0.017 (− 0.002*,* 0.036)*
A member of the military or National Guard										
Not asked the question†	2558	21.8 (20.9, 22.8)	2468	30.2 (28.9, 31.5)	2013	27.0 (25.8, 28.4)	9338	7.8 (6.3, 9.2)	8109	− 2.9 (− 4.4, − 1.3)
Not willing	9651	71.2 (70.1, 72.2)	6406	62.9 (61.5, 64.3)	6361	66.3 (64.9, 67.7)	9338	− 7.6 (− 9.2, − 6.0)	8109	2.6 (0.9, 4.3)
Somewhat willing	450	4.0 (3.5, 4.5)	272	4.0 (3.4, 4.6)	267	3.8 (3.2, 4.4)	9338	− 0.1 (− 0.9, 0.7)	8109	0.0 (− 0.8, 0.9)
Very or completely willing	180	1.9 (1.6, 2.3)	119	1.9 (1.5, 2.4)	126	2.0 (1.6, 2.5)	9338	− 0.2 (− 0.7, 0.4)	8109	0.3 (− 0.2, 0.9)
Non-response	108	1.1 (0.9, 1.4)	73	1.1 (0.8, 1.4)	71	0.9 (0.7, 1.2)	9338	0.1 (− 0.3, 0.5)	8109	− 0.1 (− 0.5, 0.2)
*Item score‡*	*10,281*	*1.10 (1.09*,* 1.11)*	*6797*	*1.11 (1.10*,* 1.13)*	*6754*	*1.11 (1.09*,* 1.12)*	*5912*	*− 0.011 (− 0.030*,* 0.008)*	*5116*	*0.012 (− 0.005*,* 0.030)*
A police officer										
Not asked the question†	2558	21.8 (20.9, 22.8)	2468	30.2 (28.9, 31.5)	2013	27.0 (25.8, 28.4)	9338	7.8 (6.3, 9.2)	8109	− 2.9 (− 4.4, − 1.3)
Not willing	9549	70.3 (69.2, 71.3)	6297	61.3 (59.9, 62.7)	6285	65.1 (63.6, 66.5)	9338	− 8.6 (− 10.2, − 7.0)	8109	2.8 (1.1, 4.5)
Somewhat willing	531	4.6 (4.1, 5.1)	342	5.1 (4.4, 5.9)	338	4.9 (4.2, 5.6)	9338	0.6 (− 0.2, 1.5)	8109	0.1 (− 0.8, 1.0)
Very or completely willing	204	2.2 (1.8, 2.6)	152	2.3 (1.9, 2.8)	143	2.2 (1.8, 2.8)	9338	0.0 (− 0.6, 0.6)	8109	0.2 (− 0.5, 0.8)
Non-response	105	1.1 (0.9, 1.4)	79	1.1 (0.9, 1.5)	59	0.8 (0.6, 1.1)	9338	0.2 (− 0.2, 0.6)	8109	− 0.2 (− 0.6, 0.1)
* Item score‡*	*10,284*	*1.12 (1.10*,* 1.13)*	*6791*	*1.14 (1.12*,* 1.16)*	*6766*	*1.13 (1.11*,* 1.15)*	*5907*	*0.009 (− 0.010*,* 0.028)*	*5119*	*0.000 (− 0.021*,* 0.021)*
A person who does not share your race or ethnicity										
Not asked the question†	2558	21.8 (20.9, 22.8)	2468	30.2 (28.9, 31.5)	2013	27.0 (25.8, 28.4)	9338	7.8 (6.3, 9.2)	8109	− 2.9 (− 4.4, − 1.3)
Not willing (1)	9865	72.8 (71.8, 73.8)	6477	63.7 (62.3, 65.1)	6454	67.5 (66.1, 68.9)	9338	− 8.5 (− 10.0, − 6.9)	8109	3.1 (1.5, 4.8)
Somewhat willing (2)	290	2.8 (2.4, 3.3)	218	3.4 (2.8, 4.0)	210	2.9 (2.4, 3.4)	9338	0.5 (− 0.2, 1.3)	8109	− 0.4 (− 1.2, 0.4)
Very or completely willing (3)	126	1.5 (1.2, 1.8)	90	1.5 (1.1, 1.9)	97	1.7 (1.3, 2.2)	9338	− 0.2 (− 0.7, 0.3)	8109	0.5 (0.0, 1.0)
Non-response	108	1.1 (0.8, 1.4)	85	1.3 (1.0, 1.7)	64	0.9 (0.6, 1.2)	9338	0.3 (− 0.1, 0.7)	8109	− 0.4 (− 0.8, 0.0)
* Item score‡*	*10,281*	*1.07 (1.06*,* 1.08)*	*6785*	*1.09 (1.08*,* 1.11)*	*6761*	*1.09 (1.07*,* 1.10)*	*5900*	*0.008 (− 0.008*,* 0.023)*	*5118*	***0.048 (0.029***,*** 0.067)***
A person who does not share your religion										
Not asked the question†	2558	21.8 (20.9, 22.8)	2468	30.2 (28.9, 31.5)	2013	27.0 (25.8, 28.4)	9338	7.8 (6.3, 9.2)	8109	− 2.9 (− 4.4, − 1.3)
Not willing (1)	9897	73.0 (72.0, 74.0)	6500	63.9 (62.6, 65.3)	6479	67.7 (66.3, 69.1)	9338	− 8.4 (− 9.9, − 6.8)	8109	3.0 (1.4, 4.7)
Somewhat willing (2)	255	2.6 (2.2, 3.1)	194	3.1 (2.5, 3.7)	177	2.6 (2.1, 3.2)	9338	0.3 (− 0.4, 1.0)	8109	− 0.1 (− 0.8, 0.6)
Very or completely willing (3)	117	1.3 (1.0, 1.6)	104	1.7 (1.3, 2.2)	103	1.8 (1.4, 2.3)	9338	0.4 (− 0.1, 0.9)	8109	0.2 (− 0.3, 0.7)
Non-response	120	1.3 (1.0, 1.6)	72	1.1 (0.8, 1.4)	66	0.9 (0.6, 1.2)	9338	− 0.1 (− 0.5, 0.3)	8109	− 0.2 (− 0.6, 0.1)
* Item score‡*	*10,269*	*1.07 (1.06*,* 1.08)*	*6798*	*1.10 (1.08*,* 1.11)*	*6759*	*1.09 (1.07*,* 1.10)*	*5903*	*0.018 (0.001*,* 0.036)*	*5121*	***0.051 (0.032***,*** 0.070)***
A person who does not share your political beliefs										
Not asked the question†	2558	21.8 (20.9, 22.8)	2468	30.2 (28.9, 31.5)	2013	27.0 (25.8, 28.4)	9338	7.8 (6.3, 9.2)	8109	− 2.9 (− 4.4, − 1.3)
Not willing (1)	9757	72.1 (71.1, 73.2)	6417	63.2 (61.9, 64.6)	6383	66.3 (64.9, 67.7)	9338	− 8.3 (− 9.9, − 6.7)	8109	2.2 (0.5, 3.8)
Somewhat willing (2)	403	3.6 (3.1, 4.1)	277	3.9 (3.3, 4.6)	265	3.9 (3.3, 4.6)	9338	0.3 (− 0.5, 1.0)	8109	0.4 (− 0.4, 1.2)
Very or completely willing (3)	119	1.3 (1.0, 1.6)	97	1.6 (1.2, 2.1)	107	1.8 (1.4, 2.3)	9338	0.2 (− 0.3, 0.7)	8109	0.4 (− 0.1, 1.0)
Non-response	110	1.2 (0.9, 1.4)	79	1.1 (0.8, 1.4)	70	0.9 (0.7, 1.3)	9338	0.1 (− 0.3, 0.5)	8109	− 0.1 (− 0.5, 0.2)
* Item score‡*	*10,279*	*1.08 (1.07*,* 1.09)*	*6791*	*1.10 (1.09*,* 1.12)*	*6755*	*1.10 (1.09*,* 1.12)*	*5909*	*0.009 (− 0.009*,* 0.027)*	*5119*	***0.042 (0.023***,*** 0.062)***

Change scores have a potential range from 0 (no change) to ±2. Change scores for 2023-2034 that differ significantly from 0 are in bold font. Findings for 2022 and 2023 respondents and mean differences for 2022-2023 were published previously [10] and are reproduced for convenience

* Among respondents to both surveys

† These respondents answered “never justified” to all prior questions on the use of force or violence to advance specific political objectives. They were not asked questions on their personal willingness to use political violence

‡ Mean scores were calculated using values indicated in the response lines for individual items. Non-responses were excluded from mean score calculations and differences in mean scores were computed in the subsample of respondents with non-missing responses in both years by computing within-individual change scores and averaging them, to account for the longitudinal study design. For computing differences in individual response levels, indicator variables were computed for each item for each response level and within-individual differences in these were computed and averaged in the subsample of respondents who responded to the survey in both years. This explains the variation in the unweighted n for the mean differences

There were no changes from 2023 to 2024 in expectations of firearm possession and use in situations where respondents considered political violence to be justified (Table 10, Fig. 1).

**Table 10:** Future likelihood of firearm possession and use in a situation where political violence is perceived as justified

Thinking now about the future and all the changes it might bring, how likely is it that you will use a gun in any of the following ways in the next few years—in a situation where you think force or violence is justified to advance an important political objective?	2022 Respondents (*n*= 12,947)		2023 Respondents (*n*=9385)		2024 Respondents (*n*=8896)		Mean Difference, * 2022-2023		Mean Difference, * 2023-2024	
	Unweighted *n*	Weighted % (95% CI) Mean score (95% CI)	Unweighted *n*	Weighted % (95% CI) Mean score (95% CI)	Unweighted *n*	Weighted % (95% CI) Mean score (95% CI)	Unweighted *n*	Weighted mean (95% CI) Mean score (95% CI)	Unweighted *n*	Weighted % (95% CI) Mean score (95% CI)
I will be armed with a gun.										
Not likely (1)	10,408	80.6 (79.7, 81.5)	6832	75.9 (74.7, 77.1)	6688	77.4 (76.2, 78.6)	9385	− 5.6 (− 6.9, − 4.4)	8185	1.0 (− 0.3, 2.3)
Somewhat likely (2)	1331	10.5 (9.8, 11.3)	1268	12.8 (11.9, 13.8)	1108	11.5 (10.6, 12.4)	9385	2.7 (1.6, 3.8)	8185	− 1.4 (− 2.6, − 0.2)
Very or extremely likely (3)	1070	7.4 (6.9, 8.0)	1140	9.0 (8.3, 9.8)	954	8.8 (8.0, 9.6)	9385	2.2 (1.4, 3.0)	8185	0.1 (− 0.8, 1.0)
Non-response	138	1.4 (1.1, 1.7)	145	2.2 (1.8, 2.7)	146	2.3 (1.9, 2.9)	9385	0.7 (0.2, 1.2)	8185	0.3 (− 0.3, 0.8)
* Item score†*	*12,809*	*1.26 (1.24*,* 1.27)*	*9240*	*1.32 (1.30*,* 1.33)*	*8750*	*1.30 (1.28*,* 1.31)*	*9181*	*0.076 (0.059*,* 0.094)*	*7984*	*− 0.011 (− 0.029*,* 0.008)*
I will carry a gun openly, so that people know I am armed.										
Not likely (1)	11,559	88.9 (88.2, 89.7)	7992	85.6 (84.6, 86.6)	7701	86.3 (85.2, 87.3)	9385	− 4.1 (− 5.2, − 3.0)	8185	0.3 (− 0.8, 1.4)
Somewhat likely (2)	751	5.6 (5.1, 6.1)	787	7.6 (6.8, 8.4)	659	6.9 (6.2, 7.6)	9385	2.4 (1.5, 3.2)	8185	− 0.8 (− 1.8, 0.1)
Very or extremely likely (3)	489	3.9 (3.5, 4.4)	451	4.4 (3.8, 5.0)	390	4.4 (3.8, 5.1)	9385	0.9 (0.2, 1.6)	8185	0.4 (− 0.4, 1.1)
Non-response	148	1.5 (1.2, 1.8)	155	2.4 (1.9, 2.9)	146	2.4 (2.0, 3.0)	9385	0.8 (0.3, 1.3)	8185	0.2 (− 0.3, 0.7)
* Item score†*	*12,799*	*1.14 (1.13*,* 1.15)*	*9230*	*1.17 (1.15*,* 1.18)*	*8750*	*1.16 (1.15*,* 1.18)*	*9162*	*0.045 (0.030*,* 0.060)*	*7980*	*0.001 (− 0.015*,* 0.016)*
I will threaten someone with a gun.										
Not likely (1)	12,570	96.3 (95.8, 96.7)	8971	93.9 (93.1, 94.7)	8498	94.0 (93.2, 94.7)	9385	− 2.2 (− 3.0, − 1.4)	8185	− 0.2 (− 1.0, 0.7)
Somewhat likely (2)	148	1.3 (1.0, 1.6)	168	2.3 (1.8, 2.8)	165	2.2 (1.8, 2.7)	9385	1.0 (0.4, 1.5)	8185	0.0 (− 0.6, 0.6)
Very or extremely likely (3)	83	0.9 (0.7, 1.2)	101	1.6 (1.2, 2.1)	89	1.5 (1.1, 2.0)	9385	0.6 (0.2, 1.1)	8185	− 0.1 (− 0.5, 0.4)
Non-response	146	1.5 (1.2, 1.8)	145	2.2 (1.7, 2.7)	144	2.3 (1.9, 2.8)	9385	0.6 (0.1, 1.1)	8185	0.2 (− 0.3, 0.7)
* Item score†*	*12,801*	*1.03 (1.03*,* 1.04)*	*9240*	*1.06 (1.05*,* 1.07)*	*8752*	*1.05 (1.04*,* 1.06)*	*9172*	*0.024 (0.014*,* 0.034)*	*7986*	*− 0.002 (− 0.012*,* 0.008)*
I will shoot someone with a gun.										
Not likely (1)	12,372	94.8 (94.3, 95.4)	8766	92.3 (91.5, 93.2)	8372	92.9 (92.0, 93.6)	9385	− 2.5 (− 3.4, − 1.6)	8185	0.2 (− 0.7, 1.1)
Somewhat likely (2)	302	2.6 (2.2, 2.9)	333	3.7 (3.1, 4.3)	264	3.2 (2.7, 3.7)	9385	1.3 (0.6, 2.0)	8185	− 0.4 (− 1.1, 0.3)
Very or extremely likely (3)	132	1.1 (0.9, 1.4)	146	1.8 (1.4, 2.2)	120	1.7 (1.3, 2.2)	9385	0.6 (0.1, 1.1)	8185	− 0.1 (− 0.6, 0.4)
Non-response	141	1.5 (1.2, 1.8)	140	2.2 (1.7, 2.6)	140	2.3 (1.9, 2.8)	9385	0.6 (0.1, 1.1)	8185	0.3 (− 0.3, 0.8)
* Item score†*	*12,806*	*1.05 (1.04*,* 1.06)*	*9245*	*1.07 (1.06*,* 1.08)*	*8756*	*1.07 (1.06*,* 1.08)*	*9179*	*0.027 (0.016*,* 0.038)*	*7993*	*− 0.005 (− 0.017*,* 0.006)*

Change scores have a potential range from 0 (no change) to ±2. Change scores for 2023-2034 that differ significantly from 0 are in bold font. Findings for 2022 and 2023 respondents and mean differences for 2022-2023 were published previously [10] and are reproduced for convenience

* Among respondents to both surveys

† Mean scores were calculated using values indicated in the response lines for individual items. Non-responses were excluded from mean score calculations and differences in mean scores were computed in the subsample of respondents with non-missing responses in both years by computing within-individual change scores and averaging them, to account for the longitudinal study design. For computing differences in individual response levels, indicator variables were computed for each item for each response level and within-individual differences in these were computed and averaged in the subsample of respondents who responded to the survey in both years. This explains the variation in the unweighted n for the mean differences

### Supplemental analyses

In 2023 and 2024, participants were asked about the justification for force or violence to advance 2 additional political objectives. There was no change in support for violence “to protect the environment or stop climate change” or “to protect the rights of animals” (Supplement, Additional File 1, Table S2).

In 2022 and 2024, respondents were asked about their personal willingness to engage in violence by the social context of that engagement (Supplement, Additional File 1, Table S3). In 2024, respondents were less likely than in 2022 to “use force or violence on your own, as an individual” (change − 0.111, 95% CI, -0.135, -0.086) and more likely to “organize a group of people who share your beliefs to use force or violence” (change 0.072, 95% CI, 0.052, 0.092).

### Sensitivity analysis

There were no differences between pre- and post-conviction respondents on any measures concerning violence; selected measures are presented in Table S4 (Supplement, Additional File 1, Table S4). Pre-conviction respondents more frequently agreed strongly or very strongly with the statement that “democracy is the best form of government,” (pre-conviction: 71.4%, 95% CI, 69.9%, 72.9%; post-conviction: 65.7%, 95% CI, 62.4%, 68.9%). There were no other differences between the groups on measures that did not concern violence.

## Discussion

From 2023 to 2024, there were few changes on measures of support for or willingness to participate in political violence in the USA, and the observed changes were small and of questionable importance. This good news runs counter to our expectation that these measures would show increases during a presidential election year associated with heightened political polarization [1]. Two findings established in Wave 1 of the survey [9] have persisted through 2023^10^ to mid-2024: the vast majority of respondents repeatedly reject political violence, and most of those who support it in principle would not want to participate in it themselves. Substantial proportions of those who currently expect to participate in violence are open to abandoning that expectation [15]. Perhaps the coupling of polarization with violence is not inevitable.

Our findings are concordant with those of other surveys from 2023 and early 2024 [31–33]. The Armed Conflict Location & Event Data (ACLED) group, which tracks instances of political violence, reports no increase as of September 8, 2024 [34]. Conditions are fluid, however. A new ACLED analysis [35, 36] emphasizes, as others have [6, 7, 37, 38], that the risk of political violence—particularly by right-wing violent extremist groups—remains substantial.

Our sensitivity analysis did not show an increase in support for political violence in the days immediately following the announcement of the Trump felony convictions. This is consistent with the findings of an as-yet unpublished survey that found no increase in support for political violence immediately following an attempt to assassinate Mr. Trump [36]. 

There are many potential explanations for the absence of an increase in support for political violence in the USA in this election year. The most prominent outbreak of political violence in the USA’s recent history, the Capitol riots of January 2021, did not achieve its objective. More than 1,000 individuals have been convicted of crimes connected with that event, and hundreds of trials are still pending [39]. The fact that participation in political violence can have significant long-term adverse consequences likely serves as a deterrent for many potential participants. Separately, 2020 was not just an election year but a time of almost unprecedented social upheaval on many fronts in the USA; 2024, up to the time of the survey, was not.

What are the implications of these findings, and others from this 2024 survey [16], for prevention? Continued public awareness of the threat posed by political violence is essential. Members of the public, community and religious leaders, elected officials, and the media should openly and repeatedly declare their rejection of political violence. They can do this with an expectation that their efforts will have an impact; from 20 to 45% of those who expect to participate in political violence say that they would change their views if urged to do to by others [16]. 

Longer-term approaches should focus on structural reform and behavior change; intervening on underlying attitudes and beliefs has disappointingly little effect [1]. Recommendations for policy and social change have been developed [40–43]. To these should be added extreme risk protection order (ERPO) laws, which allow for a temporary prohibition on the purchase and possession of firearms by people who are at high-risk of harming themselves or others. California has recently amended its law to require that judges evaluating ERPO petitions consider any evidence regarding “a recent threat of violence or act of violence directed toward another group or location, or a past history of those threats or acts.” [44] Other states should follow suit. Finally, the public should be encouraged to follow the maxim, “if you see something, say something”; many prevention measures depend on critical information about threatened violence getting to those in a position to do something about the threat [45]. 

### Limitations

Several technical limitations exist. The survey was in the field in mid-2024 and could not detect changes in support for political violence that developed later, as elections approached. The findings are subject to sampling error, inattentive or strategic responses, and nonresponse bias. Arguably, nonresponse was most important in Wave 1; the response rates for Wave 2 (84%) and Wave 3 (88%) were high. A few outcomes are uncommon, with response weighted prevalences below 5%. The large study sample and small prevalence estimates result in relatively narrow confidence intervals in these cases. This analysis presents only population-wide trends and does not examine variation among subgroups. Other analyses of data from all waves of the survey have found large subgroup differences [11–15], most notably a remarkable degree of support for and willingness to participate in political violence among supporters of organizations such as the Proud Boys and social movements such as the militia movement [32]. These subgroup differences may change over time.

External events may have affected our findings. In 2022, widely publicized mass shootings occurred in Buffalo, NY and Uvalde, TX while the survey was in the field; there were no comparable events during the fielding of the 2023 survey. The Buffalo shooting is understood to have been a race-related hate crime motivated by great replacement thinking and may have affected respondents’ views on race, violence, and that particular belief. In 2023, the survey closed just before the federal criminal indictment of Donald Trump was handed down; support for violence to return him to the White House increased immediately thereafter [46]. In 2024, the survey was in the field when convictions on those charges were announced, but our sensitivity analysis found no effect of that event on support for or willingness to participate in political violence. In all years, Russia’s war against Ukraine may have influenced responses on violence and democracy.

## Conclusions

Findings from this large, nationally representative longitudinal survey indicate that from 2023 to 2024, there was little to no change in support for or willingness to participate in political violence in the USA. This hopeful finding was contrary to our expectation, as 2024 is an election year in the USA. The findings of this analysis will be useful in designing prevention efforts.

## Electronic supplementary material

Below is the link to the electronic supplementary material.

Supplementary Material 1.

## Data Availability

The datasets generated and/or analyzed during the current study are not publicly available as analyses are continuing but will be made available to qualified researchers subject to the terms of a data use agreement.

## Abbreviations

SD: Standard deviation
CI: Confidence interval

## Declarations

Thank you very much again for responding to our Life in America survey in May or June of 2022. This new survey is about life in the United States in 2023. Like many surveys, this survey may include some questions that you might consider personal, such as questions about your thoughts on social and political issues. Your opinions and experiences are important for understanding and responding to the needs of our communities and our country. As a reminder, your responses are only ever used for research purposes and will remain anonymous – results are reported only for groups, not for individuals. Participation is voluntary, and you can choose not to answer any question. Answering the questions means that you accept us collecting the data.

It should take you about 20 min to complete the questions in this survey.

If you have any questions about this survey, you may contact the research team by calling (916) 734–3539. This study has been approved by the Institutional Review Board of the University of California, Davis. If you have any questions about your rights as a participant in this study, you may contact the University of California, Davis, Institutional Review Board at (916) 703–9151. If you have questions about your rights as a research subject or are dissatisfied at any time with any aspect of the survey, you may also contact KnowledgePanel member support at (800) 782–6899.

By continuing, you are agreeing to participate in this study.
