# Supplementary material for: Trends in views of democracy and society and support for political violence in the USA, 2022–2024: findings from a nationally representative survey
Source: Inj Epidemiol. 2025 Jan 17;12:4. doi: 10.1186/s40621-024-00550-0 (PMC11748602; doi:10.1186/s40621-024-00550-0)
Supplement: Supplementary file 1 — Supplementary Material 1. [file 40621_2024_550_MOESM1_ESM.pdf]

Supplement

Trends in Views of Democracy and Society and Support for Political Violence in the USA, 2022-2024: Findings from a Nationally Representative Survey

Garen J. Wintemute, MD, MPH; Andrew Crawford, PhD; Elizabeth A. Tomsich, PhD; Veronica A. Pear, PhD, MPH, MA

This supplement has been provided by the authors to give readers additional information about the work.

Page	Title
2	Questions that supplied data for this study
10	References for the question list
11	Additional results text
12	Figure S1. Flowchart of survey assignment and completion counts for Waves 1-3 in 2022-2024
13	Table S1. Sociodemographic characteristics of respondents and non-respondents (unweighted) in the 2022, 2023, and 2024 surveys
15	Table S2. Justification for political violence for 2 additional objectives presented in 2023 and 2024
16	Table S3. Personal willingness to engage in political violence, by social context
17	Table S4. Selected results from pre- post-conviction sensitivity analysis

QUESTIONS THAT SUPPLIED DATA FOR THIS STUDY

Response options are presented here in order from negative to positive (e.g., "not important" to "extremely important"). Respondents were randomized 1:1 to receive responses in that order or the reverse.

In the list below, questions or items that were repeated or adapted from prior surveys by other investigators contain citations to those surveys.

Domain 1: democracy in the United States

Now we'd like to ask you a few questions about the United States as you see it now, in 2024.

Q: When thinking about democracy in the United States these days, do you believe…?[1]

1. There is a serious threat to our democracy.
2. There may be a threat to our democracy, but it is not serious.
3. There is no threat to our democracy.

Q: How important do you think it is for the United States to remain a democracy?[2]

1. Not important
2. Somewhat important
3. Very important
4. Extremely important

Q: How much do you agree or disagree with the following statements about democracy in the United States?

a. Democracy is the best form of government.[3]

b. These days, American democracy only serves the interests of the wealthy and powerful.[4]

c. Having a strong leader for America is more important than having a democracy.

d. The 2020 election was stolen from Donald Trump, and Joe Biden is an illegitimate president.

1. Do not agree
2. Somewhat agree
3. Strongly agree

4. Very strongly agree

Q: People have different views about violence in the United States. How much do you agree or disagree with each of the following statements?

a. If elected leaders will not protect American democracy, the people must do it themselves, even if it requires taking violent actions.[4]

b. Because things have gotten so far off track, true American patriots may have to resort to violence in order to save our country.[5]

c. Our American way of life is disappearing so fast that we may have to use force to save it.[4]

1. Do not agree
2. Somewhat agree
3. Strongly agree
4. Very strongly agree

Q: How much do you agree or disagree with each of the following statements?

a. In the next few years, there will be civil war in the United States.[6]

1. Do not agree
2. Somewhat agree
3. Strongly agree
4. Very strongly agree

Domain 2: American society and institutions

Q: How much do you agree or disagree with each of the following statements about people in the United States today?

a. White people benefit from advantages in society that Black people do not have.[4]

b. Discrimination against whites is as big a problem as discrimination against Blacks and other minorities.[4]

c. A group of people in this country is trying to replace native-born Americans with immigrants and people of color who share their political views. (In 2022: In America, native-born white people are being replaced by immigrants.)

d. Having more Black Americans, Latinos, and Asian Americans is good for the country.[7]

1. Do not agree
2. Somewhat agree
3. Strongly agree
4. Very strongly agree

Q: People have many different views about society in the United States. How much do you agree or disagree with each of the following?

a. The government, media, and financial worlds in the U.S. are controlled by a group of Satan-worshipping pedophiles who run a global child sex trafficking operation.[5]

b. There is a storm coming soon that will sweep away the elites in power and restore the rightful leaders.[5]

c. The chaos in America today is evidence that we are living in what the Bible calls "the end times."[8]

1. Do not agree
2. Somewhat agree
3. Strongly agree
4. Very strongly agree

Domain 3: violence, including political violence

Now we have a few questions about the use of force or violence. "Force or violence" means physical force strong enough that it could cause pain or injury to a person. A reminder: your responses will be kept confidential and anonymous.

Q: People sometimes talk about using force or violence to achieve political objectives. In general, what do you think about using force or violence to advance an important political objective that you support—is it…?

1. Never justified
2. Sometimes justified
3. Usually justified
4. Always justified

Q: Your view of the use of force or violence to advance an important political objective might depend on the specific objective that was involved. What do you think about the use of force or violence in the following situations—is it never justified, sometimes justified, usually justified, or always justified?

a. To return Donald Trump to the presidency this year

b. To stop an election from being stolen

c. To stop people who do not share my beliefs from voting

d. To prevent discrimination based on race or ethnicity

e. To preserve an American way of life based on Western European traditions

f. To oppose the government when it does not share my beliefs

g. To oppose the government when it tries to take private land for public purposes

1. Never justified
2. Sometimes justified
3. Usually justified
4. Always justified

Q: You said that in general, the use of force or violence was [response inserted] to advance an important political objective that you support. Your opinion might depend on the specific objective that was involved. What do you think about the use of force or violence in the following situations—is it never justified, sometimes justified, usually justified, or always justified?

a. To stop voter fraud

b. To stop voter intimidation

c. To stop police violence

d. To reinforce the police

e. To stop illegal immigration

f. To keep our borders open

e. To stop a protest or demonstration

f. To support a protest or demonstration

g. To preserve the American way of life l believe in

h. To oppose Americans who do not share my beliefs

1. Never justified
2. Sometimes justified
3. Usually justified
4. Always justified

[Items a-f were paired in 2022, and each respondent was presented with 1 item from each pair. In 2023 and 2024 all items were presented to all respondents.]

Q: Here's another group of political objectives. What do you think about the use of force or violence in the following situations—is it never justified, sometimes justified, usually justified, or always justified?

a. To protect the environment or stop climate change

b. To protect the rights of animals

c. To stop illegal immigration

d. To keep our borders open

1. Never justified

2. Sometimes justified
3. Usually justified
4. Always justified

[Items c and d were paired in 2022, and each respondent was presented with 1 item from each pair. In 2023 and 2024 all items were presented to all respondents. Items a and b were added in 2023.]

(Questions asked of respondents who endorsed at least 1 use of violence to achieve a specific political objective.)

Q: You agreed that the use of force or violence could be justified to advance [one/some] of the political objectives we just discussed. In [that/those] [situation/situations], how willing would you personally be to…

a. Use force or violence as part of a group of people who share your beliefs

b. Use force or violence on your own, as an individual

c. Organize a group of people who share your beliefs to use force or violence

1. Not willing
2. Somewhat willing
3. Very willing
4. Completely willing

[Question asked in 2022 and 2024.]

Q: In a situation where you think force or violence is justified to advance an important political objective, how willing would you personally be to use force or violence in each of these ways?

a. To damage property

b. To threaten or intimidate a person

c. To injure a person

d. To kill a person

1. Not willing
2. Somewhat willing
3. Very willing
4. Completely willing

Q: In a situation where you think force or violence is justified to advance an important political objective, how willing would you personally be to use force or violence against a person because they are…

a. An elected federal or state government official

b. An elected local government official

c. A public health official

d. A member of the military or National Guard

e. A police officer

f. A person who does not share your race or ethnicity

g. A person who does not share your religion

h. An election worker, such as a poll worker or vote counter

i. A person who does not share your political beliefs

1. Not willing
2. Somewhat willing
3. Very willing
4. Completely willing

(Question asked of all respondents.)

Q: Thinking now about the future and all the changes it might bring, how likely is it that you will use a gun in any of the following ways in the next few years—in a situation where you think force or violence is justified to advance an important political objective?

a. I will be armed with a gun.

b. I will carry a gun openly, so that people know I am armed.

c. I will threaten someone with a gun.

d. I will shoot someone with a gun.

1. Not likely
2. Somewhat likely
3. Very likely
4. Extremely likely

REFERENCES FOR THE QUESTION LIST

1. NPR/PBS NewsHour/Marist National Poll. Trust in elections, threat to democracy, November 2021. 2021 November 1. https://maristpoll.marist.edu/polls/npr-pbs-newshour-marist-national-poll-trust-in-elections-threat-to-democracy-biden-approval-november-2021/.
2. Grinnell College National Poll. 52% of Americans believe democracy facing “major threat.” Study #2243. 2021 October 20. https://www.grinnell.edu/news/52-americans-believe-democracy-facing-major-threat.
3. The Economist/YouGov Poll. 2021 June 13-16. https://docs.cdn.yougov.com/uagnfc262c/econTabReport.pdf.
4. Survey Center on American Life. January 2021 American Perspectives Survey topline questionnaire. https://www.americansurveycenter.org/wp-content/uploads/2021/03/January-2021-APS-Topline-Questionnaire.pdf.
5. Public Religion Research Institute. The persistence of Q-Anon in the post-Trump era: an analysis of who believes the conspiracies. 2022 Feb 24. https://www.prri.org/research/the-persistence-of-qanon-in-the-post-trump-era-an-analysis-of-who-believes-the-conspiracies/.
6. Zogby. Will the US have another civil war? 2021 Feb 4. https://zogbyanalytics.com/news/997-the-zogby-poll-will-the-us-have-another-civil-war
7. Pew Research Center. Americans see advantages and challenges in country’s growing racial and ethnic diversity. 2019 May. https://www.pewresearch.org/social-trends/2019/05/08/americans-see-advantages-and-challenges-in-countrys-growing-racial-and-ethnic-diversity/.
8. IFYC – PRRI Survey on Religion & COVID-19 Vaccine Trust. 2021 March. https://www.prri.org/wp-content/uploads/2021/05/Topline-IFYC-PRRI-Survey-on-Religion-and-COVID-19-Vaccine-Trust-v2_final.pdf.

ADDITIONAL RESULTS TEXT

One item in the 2024 survey had a nonresponse percentage above 3.0%:

Q: How much do you agree or disagree with each of the following statements about people in the United States today?

Having more Black Americans, Latinos, and Asian Americans is good for the country.

Nonresponse= 3.1%.

Figure S1. Flowchart of survey assignment and completion counts for Waves 1-3 in 2022-2024

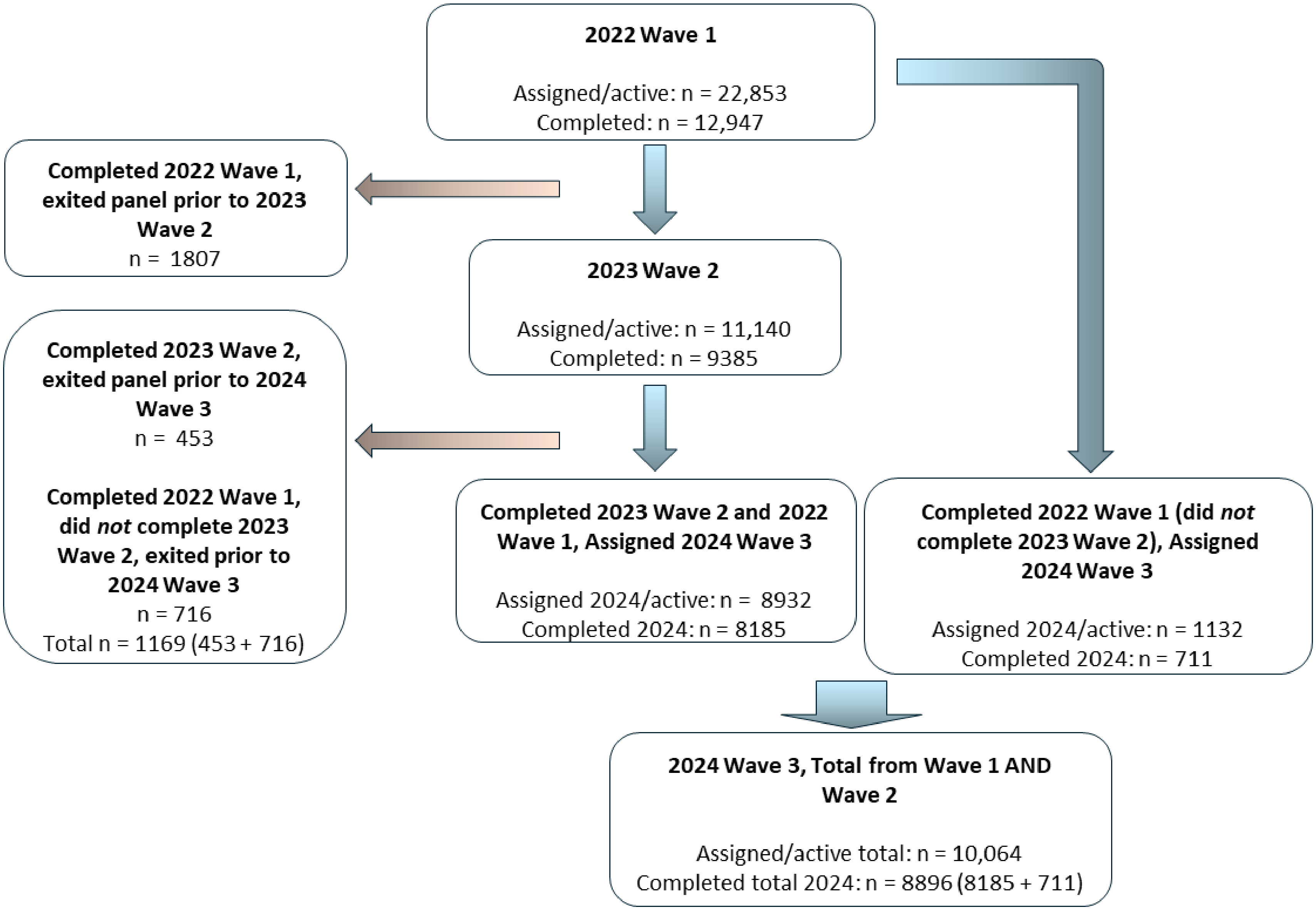

Table S1. Sociodemographic characteristics of respondents and non-respondents (unweighted) in the 2022, 2023, and 2024 surveys*

Characteristics	2022 (Wave 1)				Wave 1 respondents who left the panel prior to Wave 2 (n = 1,807)	
	Respondents* (n = 12,947)		Non-respondents (n = 9906)			
	Unweighted n	Unweighted %	Unweighted n	Unweighted %	Unweighted n	Unweighted %
**Age**						
18-24	488	3.8	1121	11.3	86	4.8
25-34	1309	10.1	1575	15.9	210	11.6
35-44	1884	14.6	1969	19.9	326	18
45-54	1847	14.3	1882	19.0	335	18.5
55-64	2794	21.6	1597	16.1	391	21.6
65-74	2952	22.8	1188	12.0	313	17.3
75+	1673	12.9	574	5.8	146	8.1
Non-response	0	0.0	0	0.0	0	0.0
**Gender**						
Male	7158	55.3	4690	47.3	854	47.3
Female	5789	44.7	5216	52.7	953	52.7
Non-response	0	0.0	0	0.0	0	0.0
**Race/Ethnicity**						
Black, non-Hispanic	1097	8.5	1039	12.5	170	9.4
Hispanic, any race	1504	11.6	1561	18.8	237	13.1
White, non-Hispanic	9493	73.3	5030	60.5	1272	70.4
Other, non-Hispanic	499	3.9	370	4.4	77	4.3
2+races, non-Hispanic	354	2.7	318	3.8	51	2.8
Non-response	0	0.0	0	0.0	0	0.0
**Marital status**						
Now married	8074	62.4	5443	54.9	1089	60.3
Widowed	770	5.9	405	4.1	82	4.5
Divorced	1456	11.2	1049	10.6	240	13.3
Separated	193	1.5	223	2.3	34	1.9
Never married	2454	19.0	2786	28.1	362	20
Non-response	0	0.0	0	0.0	0	0.0
**Education**						
No high school diploma or GED	624	4.8	754	7.6	121	6.7
High school graduate (diploma, G	2813	21.7	2410	24.3	452	25
Some college or Associate's degr	3896	30.1	3173	32.0	584	32.3
Bachelor's degree	3133	24.2	2117	21.4	372	20.6
Master’s degree or higher	2481	19.2	1452	14.7	278	15.4
Non-response	0	0.0	0	0.0	0	0.0
**Household Income**						
Less than $10,000	371	2.9	489	4.9	72	4
$10,000 to $24,999	1078	8.3	935	9.4	189	10.5
$25,000 to $49,999	2232	17.2	1829	18.5	318	17.6
$50,000 to $74,999	2236	17.3	1709	17.3	313	17.3
$75,000 to $99,999	1999	15.4	1458	14.7	236	13.1
$100,000 to $149,999	2410	18.6	1745	17.6	336	18.6
$150,000 or more	2621	20.2	1741	17.6	343	19
Non-response	0	0.0	0	0.0	0	0.0
**Employment**						
Working full-time	5645	43.6	5252	53.0	889	49.2
Working part-time	1620	12.5	1562	15.8	258	14.3
Not working	5682	43.9	3092	31.2	660	36.5
Non-response	0	0.0	0	0.0	0	0.0
**Census division**						
New England	509	3.9	371	3.7	73	4
Mid-Atlantic	1407	10.9	1068	10.8	191	10.6
East-North Central	1878	14.5	1356	13.7	262	14.5
West-North Central	952	7.4	729	7.4	137	7.6
South Atlantic	2538	19.6	1978	20.0	326	18
East-South Central	737	5.7	701	7.1	117	6.5
West-South Central	1371	10.6	1253	12.6	207	11.5
Mountain	1125	8.7	695	7.0	156	8.6
Pacific	2430	18.8	1755	17.7	338	18.7
Non-response	0	0.0	0	0.0	0	0.0

Table S1, continued.

Characteristics	2023 (Wave 2)				Wave 2 respondents who left the panel prior to Wave 3 (n = 453)		2024 (Wave 3)			
	Respondents* (n = 9,385)		Non-respondents (n = 1,755)				Respondents* (n=8896)		Non-respondents (n = 1168)	
	Unweighted n	Unweighted %	Unweighted n	Unweighted %	Unweighted n	Unweighted %	Unweighted n	Unweighted %	Unweighted n	Unweighted %
**Age**										
18-24	310	3.3	92	5.2	11	2.4	176	2.0	64	5.5
25-34	856	9.1	243	13.8	54	11.9	753	8.5	139	11.9
35-44	1252	13.3	306	17.4	50	11.0	1094	12.3	192	16.4
45-54	1255	13.4	257	14.6	63	13.9	1150	12.9	169	14.5
55-64	2043	21.8	360	20.5	100	22.1	1827	20.5	232	19.9
65-74	2342	25.0	297	16.9	96	21.2	2249	25.3	239	20.5
75+	1327	14.1	200	11.4	79	17.4	1647	18.5	133	11.4
Non-response	0	0.0	0	0.0	0	0.0	0	0.0	0	0.0
**Gender**										
Male	5437	57.9	867	49.4	261	57.6	5147	57.9	580	49.7
Female	3948	42.1	888	50.6	192	42.4	3749	42.1	588	50.3
Non-response	0	0.0	0	0.0	0	0.0	0	0.0	0	0.0
**Race/Ethnicity**										
Black, non-Hispanic	749	8.0	178	10.1	27	6.0	702	7.9	128	11.0
Hispanic, any race	1016	10.8	251	14.3	49	10.8	959	10.8	181	15.5
White, non-Hispanic	7014	74.7	1207	68.8	351	77.5	6673	75.0	778	66.6
Other, non-Hispanic	346	3.7	76	4.3	18	4.0	316	3.6	49	4.2
2+races, non-Hispanic	260	2.8	43	2.5	8	1.8	246	2.8	32	2.7
Non-response	0	0.0	0	0.0	0	0.0	0	0.0	0	0.0
**Marital status**										
Now married	5961	63.5	1024	58.3	273	60.3	5655	63.6	681	58.3
Widowed	582	6.2	106	6	36	7.9	630	7.1	83	7.1
Divorced	1010	10.8	206	11.7	61	13.5	979	11.0	117	10
Separated	122	1.3	37	2.1	5	1.1	127	1.4	17	1.5
Never married	1710	18.2	382	21.8	78	17.2	1505	16.9	270	23.1
Non-response	0	0.0	0	0.0	0	0.0	0	0.0	0	0.0
**Education**										
No high school diploma or GED	416	4.4	87	5	17	3.8	330	3.7	66	5.7
High school graduate (diploma, GED)	2002	21.3	359	20.5	121	26.7	1784	20.1	253	21.7
Some college or Associate's degree	2773	29.5	539	30.7	147	32.5	2691	30.2	354	30.3
Bachelor's degree	2337	24.9	424	24.2	85	18.8	2257	25.4	279	23.9
Master's degree or higher	1857	19.8	346	19.7	83	18.3	1834	20.6	216	18.5
Non-response	0	0.0	0	0.0	0	0.0	0	0.0	0	0.0
**Household Income**										
Less than $10,000	233	2.5	66	3.8	13	2.9	265	3	40	3.4
$10,000 to $24,999	727	7.7	162	9.2	52	11.5	609	6.8	132	11.3
$25,000 to $49,999	1617	17.2	297	16.9	89	19.6	1446	16.3	220	18.8
$50,000 to $74,999	1631	17.4	292	16.6	76	16.8	1424	16	220	18.8
$75,000 to $99,999	1499	16.0	264	15	61	13.5	1367	15.4	160	13.7
$100,000 to $149,999	1734	18.5	340	19.4	72	15.9	1799	20.2	206	17.6
$150,000 or more	1944	20.7	334	19	90	19.9	1986	22.3	190	16.3
Non-response	0	0.0	0	0.0	0	0.0	0	0.0	0	0
**Employment**										
Working full-time	3869	41.2	887	50.5	181	40	3593	40.4	525	44.9
Working part-time	1133	12.1	229	13	59	13	1038	11.7	160	13.7
Not working	4383	46.7	639	36.4	213	47	4265	47.9	483	41.4
Non-response	0	0.0	0	0.0	0	0.0	0	0.0	0	0.0
**Census division**										
New England	374	4.0	62	3.5	13	2.9	362	4.1	42	3.6
Mid-Atlantic	1001	10.7	215	12.3	46	10.2	960	10.8	148	12.7
East-North Central	1370	14.6	246	14	52	11.5	1306	14.7	178	15.2
West-North Central	676	7.2	139	7.9	44	9.7	647	7.3	72	6.2
South Atlantic	1881	20.0	331	18.9	105	23.2	1754	19.7	233	19.9
East-South Central	538	5.7	82	4.7	23	5.1	514	5.8	63	5.4
West-South Central	965	10.3	199	11.3	47	10.4	902	10.1	123	10.5
Mountain	825	8.8	144	8.2	37	8.2	796	8.9	87	7.4
Pacific	1755	18.7	337	19.2	86	19.0	1655	18.6	222	19.0
Non-response	0	0.0	0	0.0	0	0.0	0	0.0	0	0.0

Findings for 2022 and 2023 respondents were published previously[10] and are reproduced for convenience.

* Most values are as of 2022; census division values were updated for 2024; other demographics were not re-asked.

Mean [SD] ages were as follows: Wave 1 responders, 55.7 (16.7); Wave 1 non-responders, 46.9 (16.8); Wave 1 respondents who left the panel prior to Wave 2, 52.2 (16.2); Wave 2 responders, 57.0 (16.5); Wave 2 non-responders, 52.5 (17.5); Wave 3 responders, 56.8 (16.5); Wave 3 non-responders, 53.8 (17.5); Wave 2 respondents who left the panel prior to Wave 3, 57.6 (17.4).

Table S2. Justification for political violence for 2 additional objectives presented in 2023 and 2024

What do you think about the use of force or violence in the following situations?	2023 Respondents (n=9385)		2024 Respondents (n=8896)		Mean Difference,* 2023-2024	
	Unweighted n	Weighted % (95% CI) *Mean score (95% CI)*	Unweighted n	Weighted % (95% CI) *Mean score (95% CI)*	Unweighted n	Weighted % (95% CI) *Mean score (95% CI)*
To protect the environment or stop climate change†						
Never justified (1)	7495	75.7 (74.4, 77.0)	7189	78.0 (76.7, 79.2)	8109	1.7 (0.3, 3.1)
Sometimes justified (2)	1321	16.6 (15.5, 17.7)	1123	14.4 (13.4, 15.5)	8109	-1.9 (-3.2, -0.6)
Usually or always justified (3)	474	6.9 (6.2, 7.8)	475	6.7 (6.0, 7.5)	8109	0.0 (-1.0, 1.0)
Non-response	48	0.8 (0.6, 1.1)	51	0.9 (0.6, 1.3)	8109	0.2 (-0.2, 0.6)
Item score ‡	*9290*	*1.31 (1.29, 1.32)*	*8787*	*1.28 (1.26, 1.30)*	*8052*	*-0.019 (-0.039, 0.001)*
To protect the rights of animals†						
Never justified (1)	6532	67.3 (65.9, 68.6)	6198	68.8 (67.4, 70.1)	8109	1.3 (-0.2, 2.9)
Sometimes justified (2)	1945	21.6 (20.4, 22.8)	1812	20.3 (19.1, 21.4)	8109	-1.5 (-3.0, -0.1)
Usually or always justified (3)	809	10.4 (9.6, 11.4)	776	10.1 (9.2, 11.0)	8109	-0.1 (-1.2, 1.0)
Non-response	52	0.7 (0.5, 1.0)	52	0.9 (0.7, 1.3)	8109	0.3 (-0.1, 0.6)
Item score ‡	*9286*	*1.43 (1.41, 1.45)*	*8786*	*1.41 (1.39, 1.43)*	*8045*	*-0.014 (-0.036, 0.008)*

Change scores have a potential range from 0 (no change) to ±2. Change scores for 2023-2034 that differ significantly from 0 are in bold font.

* Among respondents to both surveys

† In each year, participants who did not answer the question "In general…to advance an important political objective that you support" were not asked this question.

‡ Mean scores were calculated using values indicated in the response lines for individual items. Non-responses were excluded from mean score calculations and differences in mean scores were computed in the subsample of respondents with non-missing responses in both years by computing within-individual change scores and averaging them, to account for the longitudinal study design. For computing differences in individual response levels, indicator variables were computed for each item for each response level and within-individual differences in these were computed and averaged in the subsample of respondents who responded to the survey in both years. This explains the variation in the unweighted n for the mean differences.

Table S3. Personal willingness to engage in political violence, by social context

Here's another group of political objectives. What do you think about the use of force or violence in the following situations?	2022 Respondents (n= 12,947)		2024 Respondents (n=8896)		Mean Difference,* 2022, 2024	
	Unweighted n	Weighted % (95% CI) *Mean score (95% CI)*	Unweighted n	Weighted % (95% CI) *Mean score (95% CI)*	Unweighted n	Weighted % (95% CI) *Mean score (95% CI)*
Use force or violence as part of a group of people who share your beliefs						
Not asked the question†	2558	21.8 (20.9, 22.8)	2013	27.0 (25.8, 28.4)	8838	4.9 (3.4, 6.5)
Not willing (1)	8489	63.5 (62.4, 64.6)	5548	58.6 (57.2, 60.0)	8838	-5.0 (-6.8, -3.2)
Somewhat willing (2)	1510	11.1 (10.4, 11.9)	1042	11.1 (10.2, 12.1)	8838	-0.1 (-1.2, 1.0)
Very or completely willing (3)	306	2.6 (2.3, 3.0)	178	2.4 (2.0, 2.9)	8838	0.0 (-0.6, 0.6)
Non-response	84	0.9 (0.7, 1.1)	57	0.8 (0.6, 1.2)	8838	0.2 (-0.2, 0.5)
Item score ‡	*8838*	*0.49 (0.45, 0.53)*	*6768*	*1.22 (1.20, 1.24)*	*5829*	*0.007 (-0.015, 0.028)*
Use force or violence on your own, as an individual						
Not asked the question†	2558	21.8 (20.9, 22.8)	2013	27.0 (25.8, 28.4)	8838	4.9 (3.4, 6.5)
Not willing (1)	7534	56.9 (55.8, 58.0)	5199	55.5 (54.1, 57.0)	8838	1.6 (-0.2, 3.4)
Somewhat willing (2)	2158	15.8 (15.0, 16.6)	1262	12.7 (11.8, 13.7)	8838	-4.7 (-6.0, -3.5)
Very or completely willing (3)	621	4.7 (4.2, 5.2)	308	3.8 (3.3, 4.5)	8838	-2.1 (-2.8, -1.4)
Non-response	76	0.8 (0.6, 1.1)	56	0.9 (0.6, 1.2)	8838	0.3 (0.0, 0.6)
Item score ‡	*8843*	*0.58 (0.53, 0.62)*	*6769*	*1.28 (1.26, 1.30)*	*5837*	***-0.111 (-0.135, -0.086)***
Organize a group of people who share your beliefs to use force or violence						
Not asked the question†	2558	21.8 (20.9, 22.8)	2013	27.0 (25.8, 28.4)	8838	4.9 (3.4, 6.5)
Not willing (1)	9131	67.5 (66.5, 68.6)	6034	63.1 (61.7, 64.5)	8838	-9.1 (-10.8, -7.3)
Somewhat willing (2)	936	7.4 (6.8, 8.1)	601	6.9 (6.1, 7.7)	8838	3.6 (2.6, 4.7)
Very or completely willing (3)	251	2.4 (2.0, 2.8)	143	2.2 (1.8, 2.7)	8838	0.2 (-0.3, 0.8)
Non-response	71	0.8 (0.6, 1.1)	47	0.7 (0.5, 1.1)	8838	0.2 (-0.1, 0.6)
Item score ‡	*8845*	*0.45 (0.41, 0.49)*	*6778*	*1.16 (1.14, 1.17)*	*5837*	***0.072 (0.052, 0.092)***

Change scores have a potential range from 0 (no change) to ±2. Change scores for 2023-2034 that differ significantly from 0 are in bold font.

* Among respondents to both surveys

† These respondents answered "never justified" to all prior questions on the use of force or violence to advance specific political objectives. They were not asked questions on their personal willingness to use political violence.

‡ Mean scores were calculated using values indicated in the response lines for individual items. Non-responses were excluded from mean score calculations and differences in mean scores were computed in the subsample of respondents with non-missing responses in both years by computing within-individual change scores and averaging them, to account for the longitudinal study design. For computing differences in individual response levels, indicator variables were computed for each item for each response level and within-individual differences in these were computed and averaged in the subsample of respondents who responded to the survey in both years. This explains the variation in the unweighted n for the mean differences.

Table S4. Selected results from pre- post-conviction sensitivity analysis

Measure	Pre-Conviction		Post-Conviction	
	Unweighted n	Weighted% (95% CI)	Unweighted n	Weighted% (95% CI)
**Violence to advance political objectives**				
Violence is usually or always justified to advance at least 1 of 17 objectives*	4925	30.6 (29.1, 32.0)	1137	27.9 (25.2, 30.7)
**Specific objectives**				
To return Donald Trump to the presidency this year†				
Never justified	6518	89.5 (88.4, 90.6)	1459	89.3 (87.1, 91.2)
Sometimes justified	307	5.8 (5.0, 6.8)	88	6.2 (4.8, 8.1)
Usually or always justified	326	4.6 (4.0, 5.4)	74	4.5 (3.3, 6.0)
To threaten or intimidate a person†				
Not willing	4867	85.9 (84.5, 87.3)	1087	86.1 (83.2, 88.6)
Somewhat willing	542	10.8 (9.6, 12.1)	121	10.8 (8.6, 13.5)
Very or completely willing	126	3.3 (2.6, 4.2)	35	3.1 (2.0, 4.6)
To kill a person†				
Not willing	5171	92.0 (90.8, 93.1)	1146	91.7 (89.3, 93.6)
Somewhat willing	246	5.1 (4.3, 6.1)	72	5.9 (4.4, 8.0)
Very or completely willing	111	2.8 (2.2, 3.7)	25	2.4 (1.5, 3.9)
**Future firearm possession and use**				
I will be armed with a gun				
Not likely	5473	79.5 (78.1, 80.7)	1215	78.6 (75.9, 81.1)
Somewhat likely	885	11.5 (10.5, 12.6)	223	12.6 (10.6, 14.9)
Very or extremely likely	777	9.0 (8.1, 9.9)	177	8.8 (7.3, 10.6)
I will shoot someone with a gun				
Not likely	6833	95.1 (94.2, 95.8)	1539	94.9 (93.1, 96.2)
Somewhat likely	213	3.1 (2.6, 3.8)	51	3.6 (2.4, 5.2)
Very or extremely likely	92	1.8 (1.3, 2.4)	28	1.5 (0.9, 2.5)
**Views of democracy**				
Democracy is the best form of government				
Do not agree	383	7.0 (6.1, 7.9)	110	8.6 (6.8, 10.8)
Somewhat agree	1186	21.6 (20.2, 23.1)	321	25.7 (22.8, 28.8)
Strongly or very strongly agree	5587	71.4 (69.9, 72.9)	1188	65.7 (62.4, 68.9)

* Restricted for each respondent to the 13 items presented to that respondent in all 3 years.

† In each year, participants who did not answer the question "In general…to advance an important political objective that you support" were not asked this question.
